# Supplementary material for: Specialised orthotic care to improve functioning in adults with neuromuscular disorders: protocol of a prospective randomised open-label blinded end-point study
Source: BMJ Open. 2020 Nov 19;10(11):e039683. doi: 10.1136/bmjopen-2020-039683 (PMC7678344; doi:10.1136/bmjopen-2020-039683)
Supplement: Supplementary data [file bmjopen-2020-039683supp002.pdf]

Subject information

Supplementary file 2: consent form subject

‘Specialized orthotic care for people with leg muscle weakness’

- I have read the information letter. I was also able to ask questions. My questions have been answered sufficiently. I have had enough time to decide whether or not to participate.

- I understand that participation is voluntary. I also know that I may decide at any time to not participate or to stop participating in the study. Without having to provide any reason.

- I give consent for my general practitioner to be informed of my participation in this study.

- I give consent to information being requested from my specialist(s) treating me about my medical history.

- I give consent to collect and use my data for answering the research question in this study.

- I know that for study monitoring purposes some individuals could have access to all my data. Those people are listed in this information letter. I consent to that access by these persons.

- I give consent for my general practitioner and/or treating specialist to be informed of unexpected findings which are (may be) of interest for my health.

- I □ **give**
  □ **do not give**
  consent for the further storage of my personal data for 15 years and retention for future research into the area of my disorder and the method of treatment.

- I □ **give**
  □ **do not give**
  consent to being contacted again after this study for a follow-up study.

Subject information

- I want to participate in this study.

Name of subject: ……………………………………………………………………………..
Signature: Date : __ / __ / __

I certify that I have fully informed this subject about the said study.

If information becomes known during the study that could influence the consent of the subject, I will inform him/her of this on time.

Name of investigator :…………………………………………………………………………
Signature: Date: __ / __ / __

The subject will receive a complete information letter, together with a signed version of the informed consent form.

van Duijnhoven E, *et al. BMJ Open* 2020; 10:e039683. doi: 10.1136/bmjopen-2020-039683
