# Supplementary material for: Specialised orthotic care to improve functioning in adults with neuromuscular disorders: protocol of a prospective randomised open-label blinded end-point study
Source: BMJ Open. 2020 Nov 19;10(11):e039683. doi: 10.1136/bmjopen-2020-039683 (PMC7678344; doi:10.1136/bmjopen-2020-039683)
Supplement: Author's manuscript [file bmjopen-2020-039683.draft_revisions.pdf]

BMJ Open

BMJ Open is committed to open peer review. As part of this commitment we make the peer review history of every article we publish publicly available.

When an article is published we post the peer reviewers' comments and the authors' responses online. We also post the versions of the paper that were used during peer review. These are the versions that the peer review comments apply to.

The versions of the paper that follow are the versions that were submitted during the peer review process. They are not the versions of record or the final published versions. They should not be cited or distributed as the published version of this manuscript.

BMJ Open is an open access journal and the full, final, typeset and author-corrected version of record of the manuscript is available on our site with no access controls, subscription charges or pay-per-view fees (http://bmjopen.bmj.com).

If you have any questions on BMJ Open's open peer review process please email info.bmjopen@bmj.com

BMJ Open

Specialized orthotic care to improve functioning in adults with neuromuscular diseases: protocol of a prospective randomized open label blinded end-point study.

Journal:	*BMJ Open*
Manuscript ID	bmjopen-2020-039683
Article Type:	Protocol
Date Submitted by the Author:	22-Apr-2020
Complete List of Authors:	van Duijnhoven, Elza; Amsterdam UMC, University of Amsterdam, Department of Rehabilitation Medicine, Amsterdam Movement Sciences<br>Koopman, Fieke; Amsterdam UMC, University of Amsterdam, Department of Rehabilitation Medicine, Amsterdam Movement Sciences<br>Tuijtelaars, Jana; Amsterdam UMC, University of Amsterdam, Department of Rehabilitation Medicine, Amsterdam Movement Sciences<br>Altmann, Viola; Klimmendaal, Rehabilitation center<br>Lagrand, Rimke; Amsterdam UMC, University of Amsterdam, Department of Rehabilitation Medicine, Amsterdam Movement Sciences<br>van Dongen, Johanna; Vrije Universiteit Amsterdam, Department of Health Sciences, Faculty of Science<br>Nollet, Frans; Amsterdam UMC, University of Amsterdam, Department of Rehabilitation Medicine, Amsterdam Movement Sciences<br>Brehm, Merel-Anne; Amsterdam UMC, University of Amsterdam, Department of Rehabilitation Medicine, Amsterdam Movement Sciences
Keywords:	Neuromuscular disease < NEUROLOGY, REHABILITATION MEDICINE, Protocols & guidelines < HEALTH SERVICES ADMINISTRATION & MANAGEMENT

SCHOLARONE™
Manuscripts

I, the Submitting Author has the right to grant and does grant on behalf of all authors of the Work (as defined in the below author licence), an exclusive licence and/or a non-exclusive licence for contributions from authors who are: i) UK Crown employees; ii) where BMJ has agreed a CC-BY licence shall apply, and/or iii) in accordance with the terms applicable for US Federal Government officers or employees acting as part of their official duties; on a worldwide, perpetual, irrevocable, royalty-free basis to BMJ Publishing Group Ltd ("BMJ") its licensees and where the relevant Journal is co-owned by BMJ to the co-owners of the Journal, to publish the Work in this journal and any other BMJ products and to exploit all rights, as set out in our licence.

The Submitting Author accepts and understands that any supply made under these terms is made by BMJ to the Submitting Author unless you are acting as an employee on behalf of your employer or a postgraduate student of an affiliated institution which is paying any applicable article publishing charge ("APC") for Open Access articles. Where the Submitting Author wishes to make the Work available on an Open Access basis (and intends to pay the relevant APC), the terms of reuse of such Open Access shall be governed by a Creative Commons licence – details of these licences and which Creative Commons licence will apply to this Work are set out in our licence referred to above.

Other than as permitted in any relevant BMJ Author's Self Archiving Policies, I confirm this Work has not been accepted for publication elsewhere, is not being considered for publication elsewhere and does not duplicate material already published. I confirm all authors consent to publication of this Work and authorise the granting of this licence.

For peer review only

Specialized orthotic care to improve functioning in adults with neuromuscular diseases: protocol of a prospective randomized open label blinded end-point study.

Elza van Duijnhoven[1], Fieke Sophia Koopman[1], Jana Tuijtelaars[1], Viola Altmann[2], Rimke Lagrand[1], Johanna Maria van Dongen[3], Frans Nollet[1], Merel-Anne Brehm[1]

[1]*Amsterdam UMC, University of Amsterdam, Department of Rehabilitation Medicine, Amsterdam Movement Sciences, Meibergdreef 9, Amsterdam, the Netherlands*

[2]*Klimmendaal, Rehabilitation center, Arnhem, the Netherlands.*

[3]*Department of Health Sciences, Faculty of Science, Vrije Universiteit Amsterdam ,Amsterdam Movement Sciences, Amsterdam, the Netherlands*

*Correspondence to: Elza van Duijnhoven

Department of Rehabilitation Medicine

Amsterdam UMC, location Academic Medical Center

Meibergdreef 9

1105 AZ, Amsterdam

The Netherlands

Email: e.vanduijnhoven@amsterdamumc.nl

Telephone: +31 20 566 6915

Fax: +31 20 5669154

Issue date: 22-04-2020

Protocol amendment number: 6

Word count: 3882

For peer review only

ABSTRACT

Introduction

People suffering from leg muscle weakness caused by neuromuscular diseases (NMD) are often provided with leg orthoses to reduce walking problems such as increased walking effort, diminished walking speed, reduced balance and falls. However, evidence for the effectiveness of leg orthoses to improve walking in this patient group is limited and there is an absence of standardized practice in orthotic prescription. In 2012 a Dutch multidisciplinary guideline was developed aimed to standardize the orthotic treatment process in NMD. Although application of the guideline (specialized orthotic care) seems beneficial regarding clinical effectiveness, larger studies are necessary to confirm results and investigate cost-effectiveness. Therefore, this study aims to examine the effectiveness and cost-effectiveness of specialized orthotic care compared to usual orthotic care in adults with slowly progressive NMD.

Methods and analysis

A prospective randomized open label blinded end-point study will be performed, in which 70 adults with slowly progressive NMD are randomly assigned to specialized orthotic care (intervention) or usual orthotic care (control). Outcome measures are assessed at baseline and at 3 and 6 months follow-up. The primary endpoints are gross walking energy cost (J/kg/m) assessed during a 6-minute walk test and achievement of personal goals, measured with the goal attainment scale. Secondary endpoints include walking speed, gait biomechanics, stability, physical functioning, falls and fear of falling, perceived fatigue, and satisfaction. For the economic evaluation, societal costs and health-related quality of life will be assessed using cost questionnaires and the EQ-5D-5L, retrospectively.

Ethics and dissemination

The study is registered in the Dutch trial register (NL 7511) and the protocol has been approved by the Medical Ethics Committee of the Academic Medical Center in Amsterdam. This study will provide evidence on the (cost-)effectiveness of specialized orthotic care in adults with NMD, necessary for the improvement of quality and efficiency of orthotic care.

Keywords

Neuromuscular diseases; orthotic devices; specialized orthotic care; cost-effectiveness, walking energy cost, goal attainment scale; RCT.

For peer review only

ARTICLE SUMMARY

Strengths and limitations of this study

- A broad range of outcome measures, both objective and patient-reported, will be collected before and after orthotic treatment, resulting in a unique dataset of both functional effects, underlying biomechanical working mechanisms, and costs.
- An economic evaluation will be performed alongside the study to provide insights into the cost-effectiveness of specialized orthotic care versus usual orthotic care.
- A possible limitation is the expected variability in treatment components of usual orthotic care, which could complicate the identification of underlying mechanisms of action between treatments. The process of usual orthotic care will therefore be extensively documented.

For peer review only

INTRODUCTION

People with slowly progressive neuromuscular diseases (NMD), such as post-polio syndrome and Charcot Marie Tooth disease, frequently suffer from leg muscle weakness[1]. Leg muscle weakness will change the gait pattern[2], causing walking problems such as increased walking effort[3 4], diminished walking speed[3 4], pain[5], balance problems and falls[6 7]. These walking problems may restrict the patients' performance of daily physical activities[8] and negatively affect their independency and quality of life[8 9]. Due to the progressive nature of NMD, walking problems will gradually increase over time[9], which will further negatively affect physical mobility, independency and quality of life.

Leg orthoses are provided to reduce walking problems in people with NMD[10]. A leg orthosis is an "externally applied medical device encompassing (part of) the leg and foot, used to modify the structural and functional characteristics of the neuromuscular and skeletal systems"[11]. In general, an ankle foot orthosis (AFO) is provided in case of distal leg muscle weakness, which often includes weakness of the foot dorsiflexors and plantarflexors[10 12]. Accordingly, the AFO should support foot clearance during swing by restricting plantar flexion and compensate for plantarflexor weakness by restricting dorsiflexion during late stance[10]. Knee-ankle-foot orthoses (KAFOs) are provided for (additional) proximal leg muscle weakness, particularly weakness of the quadriceps, to stabilize the knee joint during the stance phase of gait and allow safe weight-bearing[10 13].

In previous research, various types of AFOs for calf muscle weakness[14] and KAFOs for quadriceps weakness have been found to improve walking in people with NMD[15]. However, the strength of the evidence is rather limited due to a lack of proper study designs, relatively small sample sizes, and heterogeneity in types of leg orthoses and control conditions studied. Accordingly, there is a lack of evidence-based and standardized guidance on the application of leg orthoses. Furthermore, many different types of leg orthoses are being prescribed in current orthotic practice in NMD, varying largely in orthotic properties and effectiveness, with both good and suboptimal treatment outcomes in terms of reducing walking problems[16 17].

In 2012, a Dutch multidisciplinary guideline for leg orthoses in adults with slowly progressive NMD was developed with the aim to standardize the treatment process and improve treatment outcomes[18]. The guideline was developed according to proposed national and international frameworks[19 20 21] and compromises the entire process of orthotic treatment in systematically divided steps, as well as treatment algorithms for the selection of leg orthoses. According to the guideline, the care need should be individually characterized in terms of the personal health problems, goals and gait deviations to be addressed, caused by the underlying impairments. Based on the care need, the orthotic goals should be defined and matched with the orthosis design. Gait training after delivery of the orthosis after is essential to maximize effectiveness[22]. Finally, it is important to systematically evaluate the effectiveness as well as user experiences to ensure the functionality of the orthosis[23].

At this moment, the guideline is not widely applied throughout the Netherlands, as it requires sufficient expertise of the multidisciplinary care team involved and facilities for advanced 3D-gait analysis and gait training. However, prescribing leg orthoses according to the guideline (i.e. specialized orthotic care) seems promising in terms of clinical effectiveness as recently shown in two uncontrolled trials[16 24]. In individuals with calf muscle weakness due to NMD, individually stiffness-optimized AFOs have been shown to reduce walking effort to a much greater extent compared to AFOs prescribed in usual orthotic care[24]. Furthermore, in polio survivors[16], the increment in walking effort with KAFOs

For peer review only - http://bmjopen.bmj.com/site/about/guidelines.xhtml

prescribed in specialized orthotic care was reduced with 18% towards normative values, when compared to usual care KAFOs. At this moment, the effectiveness of leg orthoses prescribed within specialized orthotic care on functioning needs to be investigated in a larger group of individuals with slowly progressive NMD in comparison to usual orthotic care to strengthen evidence for the possible benefit.

While good-quality studies are needed to strengthen the evidence for the effectiveness of specialized orthotic care in NMD, it is also imperative to investigate whether the treatment is cost-effective, which is currently unknown. Since new and expensive orthotic devices increasingly become available, it is important to assess their associated costs[25]. Also, at this moment, an important problem in usual orthotic care concerns the low compliance of patients wearing their devices[26], which in turn leads to an inefficient use of healthcare resources[27]. Optimally matching orthoses with the personal needs and goals of the patient, according to the guideline, could lead to gains in clinical effectiveness and subsequently a higher compliance. In this respect, specialized orthotic care could improve the efficiency of (healthcare) resource, but this has not yet been investigated.

The aim of this study is to examine the effectiveness and cost-effectiveness of specialized orthotic care compared to usual orthotic care on functioning in adults with NMD. We hypothesize that specialized orthotic care is more effective in terms of reducing walking effort and achieving personal goals when compared to usual orthotic care. Furthermore, specialized orthotic care is expected to be cost-effective compared with usual orthotic care from a societal and healthcare perspective.

For peer review only

METHODS AND ANALYSIS

Study design

The study is designed as a randomized open label blinded end-point study that is prospectively registered at the Dutch Trial Register under number NL7511. The trial protocol was developed according to the Standardized Protocol[28]. Measurements are conducted at baseline (T1) and 3 and 6 months after orthotic treatment is given (T2 and T3, respectively). To determine the cost-effectiveness, an economic evaluation is performed alongside the study. An overview of the study design is shown in Figure 1.

Study population

We intend to include 70 adults with slowly progressive NMD (such as Charcot-Marie-Tooth disease, post-polio syndrome, inclusion body myositis or myotonic dystrophy) or peripheral nerve injury who have non-spastic leg muscle weakness. Patients will be recruited from rehabilitation centers and hospitals throughout the Netherlands and through the Dutch Association for Neuromuscular Diseases. If a patient is willing to participate, a screening visit is planned. During the screening visit, written informed consent is obtained and the investigator, in close collaboration with a rehabilitation physician, will check the in- and exclusion criteria.

Inclusion criteria are: (1) minimum age of 18 years, (2) weakness of the calf muscles (i.e. Medical Research Council (MRC) scale[29] score < 5 or not being able to make a heel-rise on one leg > 3 times) and/or weakness of the quadriceps (i.e. MRC score < 5) (3) experiencing walking problems such as increased walking effort, pain and/or impaired balance during standing and/or walking, (4) able to walk for 6 minutes at comfortable speed with or without assistive device (e.g. cane, crutch, walker), (5) indicated for an orthosis based upon physical examination and 3D gait analysis, and (6) motivated to use an orthosis. An exclusion criterion is: (1) insufficient mastery of the Dutch language.

Sample size

The sample size of this study is based on a power analysis of the expected differences in walking effort (defined as gross walking energy cost) and the achievement of personal treatment goals between the intervention and control group. According to previous studies[16 17], a difference in change in walking energy cost of 0.60 J/kg/m from baseline to 6 months post-treatment is expected between usual orthotic care and specialized orthotic care, where a difference of at least 0.45 J/kg/m is considered as clinical relevant[3]. Based on an intention-to-treat analysis, alpha of 0.05, power of 80%, and using an estimated correlation coefficient of the repeated measures of 0.76 and standard deviation of 0.90 J/kg/m, 30 patients per treatment group are necessary. The goal is to include 70 patients allowing for a 15% drop out. This sample size also allows the detection of 1 point difference in personal achievement goal scores (Based on an intention-to-treat analysis, alpha of 0.05, power of 80%, and using an estimated correlation coefficient of the repeated measures of 0.70).

Randomization and blinding

After baseline measurement (T1), participants will be randomly assigned in a 1:1 ratio to receive specialized orthotic care (intervention group) or usual orthotic care (control group). The randomization scheme will be computer-generated in Castor (Castor EDC, Amsterdam, the Netherlands) and uses blocks of random sequences with variable sizes (2, 4 and 6). Patients will be stratified by disease

For peer review only

severity defined as distal leg weakness versus (distal and) proximal leg weakness, respectively. Outcomes will be assessed by a blinded and independent assessor. When patients are informed of group allocation, they will be instructed not to reveal this to the outcome assessor.

Intervention

Specialized orthotic care

Patients in the intervention group will receive specialized orthotic care at an expert center that has implemented the Dutch guideline for leg orthoses[18] and has facilities for 3D gait analysis, gait training and fabricating custom-made leg orthoses. For the orthotic care process, protocols of the guideline will be used and treatment integrity checks will be performed to ensure compliance with the guideline.

First, the health problems and goals of the patient will assessed by means of an interview, questionnaires, physical examination and 3D gait analysis. Based on these assessments, the multidisciplinary care team will identify how the patients' problems and goals are best matched to an orthotic solution. The desired level of functioning for the patient and the required orthotic functions will be described in a care plan. Accordingly, the orthosis will be custom fabricated by an orthotic technician. After delivery of the orthosis, instructions on use and maintenance will be given to the patient and, when indicated, the patient receives gait training supervised by an experienced physiotherapist. Finally, after 3 months of using the orthosis, experiences of the patient as well as the desired level of functioning and orthotic functions will be evaluated. When needed, the orthosis will be adjusted based on the evaluation. The provision process will be extensively documented for each participant individually by an investigator who is not blinded.

Usual orthotic care

Participants in the control group will receive usual orthotic care from a rehabilitation physician at their regular care center. Treatment can vary across centers and may include provision of orthopedic shoes, provision of off-the-shelf or custom-made AFOs or provision of off-the-shelf or custom-made KAFOs according to the discretion of the practitioners and local policy of the center. The provision process in the usual care centers will be extensively documented for each participant individually by an investigator who is not blinded.

Outcome measures

Relevant demographic variables, anthropometrics and clinical characteristics will be measured at baseline (T1). Clinical characteristics to be evaluated include; 1) manually assessed muscle strength of the left and right hip flexors and extensors, hip abductors and adductors, knee flexors and extensors, ankle plantar and dorsal flexors and ankle invertors and evertors, scored according to the MRC scale, and 2) passive range of motion of the hip, knee and ankle joints (left and right). Furthermore, 3) occurrence of joint deformities and 4) impairments in sensory function will be evaluated.

Primary outcomes (described below) and secondary outcomes will be assessed at baseline (T1) and at 3 and 6 months after orthotic treatment (T2 and T3, respectively). Additionally, orthotic mechanical properties (such as type, weight, material and orthotic functions) and adverse events due to the use of the device (such as pressure sores, pain, muscle soreness) will be documented. All collected outcomes will be entered into a Castor database. An overview of all outcomes per measurement visit is given in Table 1.

For peer review only - http://bmjopen.bmj.com/site/about/guidelines.xhtml

***Table 1**: Overview of outcomes per measurement session.*

		Baseline	Follow-up	
		**T1 measurement** *screening*	**T2 measurement** *(12 weeks after delivery)*	**T3 measurement** *(24 weeks after delivery)*
**Primary outcomes**				
Walking energy cost	*6MWT*	X	X	X
Personal goals	*GAS*	Setting goals	X	X
**Secondary outcomes**				
Walking speed	*6MWT*	X	X	X
Gait biomechanics	*3DGA*	X	X	X
Stability	*NRS*	X	X	X
Physical functioning	*SF36-PF*	X	X	X
Fear of falling	*FES*	X	X	X
Fall rate	*Questionnaire*	X	X	X
Fatigue	*FSS*	X	X	X
Satisfaction	*D-Quest*	X*	X	X
**Additional outcomes**				
Demographics	*Intake*	X		
Anthropometrics	*Physical exam*	X		
Muscle strength	*Physical exam*	X		
Joint passive range of motion	*Physical exam*	X		
Sensory function	*Physical exam*	X		
Orthotic properties	*CRF*	X*	X	
Adverse events	*CRF*		X	X
**Economic evaluation**				
Resource use**	*Cost questionnaire*	X	X	X
Health-related quality of life**	*EQ-5D-5L*	X	X	X

*Outcomes will only be assessed in case a participant uses an orthosis at baseline.
**Outcomes for the economic evaluation will also be assessed directly after delivery of the orthosis.

6MWT = 6-minute walk test, GAS = Goal Attainment Scale, 3DGA = 3-dimensional gait analysis, NRS = numeric rating scale, SF36-PF = physical functioning scale of the SF36, FES = Falls efficacy scale, FSS = Fatigue severity scale, D-Quest = Dutch version of the Quebec User Evaluation of Satisfaction with Assistive Technology, CRF = clinical report form, EQ-5D-5L = 5-level version of EuroQol 5D.

Primary outcomes

Primary outcomes are walking energy cost and the achievement of personal treatment goals. Walking energy cost will be determined during a 6-minute walk test (6MWT) in which participants walk at a self-selected comfortable speed on an indoor oval track. Simultaneously, oxygen uptake (VO_2) and the respiratory exchange ratio (RER) will be measured breath-by-breath with the Cosmed K5 portable gas analysis system (Cosmed, Rome, Italy). Mean VO_2, RER and walking speed values will be obtained at steady state from the last 3 minutes of the 6MWT using a custom-written Matlab script (V.2019; MathWorks, Natick, Massachusetts, USA). From these outcomes, walking energy cost in J/kg/m will be calculated by using the following formula: $((4.940 \times RER) + 16.040) \times VO_2)/$ walking speed where VO_2 is in ml/kg/min[30]. The assessment of walking energy cost has previously been shown to be reliable in patients with NMD[31 32].

The achievement of personal treatment goals will be quantified with the Goal Attainment Scale (GAS) to capture the diversity of orthotic treatment goals important to the individual patient[33]. At baseline, two goals that are highly relevant to the individual will be determined with the patient based on what they wish to achieve in daily life in terms of activities and participation according to the International Classification of Functioning, Disability and Health (ICF) framework[20]. Subsequently, personal GAS scales will be determined by creating six distinct levels of outcome ranging from -3 to +2, where the desired attainment goal of the patient is defined as 0 and the current situation is defined as -2. Achievement of the goals will be scored at T2 and T3 as -3 = worsened, -2 = unchanged, -1 = somewhat less than expected, 0 = expected outcome, +1 = somewhat more than expected and +2 = much more than expected. Improvements of at least 2 points will be regarded as clinical relevant[34]. The investigator defining and scoring the GAS, followed a course in applying GAS within a rehabilitation context[35].

Secondary outcomes

Secondary outcomes include walking speed (measured during the 6MWT), gait biomechanics (explained below), perceived stability during walking (assessed with a 11-point numeric rating scale (NRS)), perceived physical functioning (assessed with the Short-Form Health Survey physical functioning scale (SF36-PF)[36]), frequency of falls and fear of falling (assessed with the short version of the Falls Efficacy Scale (FES)[37], perceived fatigue (assessed with the Fatigue Severity Scale (FSS)[38]) and satisfaction with the orthosis (assessed with the Dutch version of the Quebec User Evaluation of Satisfaction with Assistive Technology (D-QUEST)[39] added with self-designed items).

Gait biomechanics

Gait biomechanics will be assessed with a 100 Hz 12-camera 3D motion capture system (VICON MX 1.3) and two adjacent 1000 Hz force plates (OR6-7; AMTI, Watertown, Marssachusetts, USA). Preparations include placement of reflective markers on the body according to the Plug-In Gait model. After a static calibration, participants will be asked to walk along a 12-m long walkway. Conditions that will be assessed include barefoot walking (T1), walking with shoes (T1), walking with the old orthosis if applicable (T1) and walking with the new orthosis (T2 and T3). Per condition, at least three valid trials will be used for analysis containing a clear stance phase on the force plate for both feet and full visibility of all markers during the gait cycle. Joint angles, net joint moments and joint powers around the hip, knee and ankle will be calculated per trial and averaged over three trials. Spatiotemporal gait parameters (such as step length and step width) and relevant kinematic and kinetic variables (such as maximal ankle dorsiflexion angle, peak ankle power, knee angle and knee moment at midstance, hip flexion angle and maximal hip, knee and ankle angle during swing and progression of the center of pressure) will be obtained from these averaged data and used for analysis.

Economic evaluation

The economic evaluation will be performed from a societal and a healthcare perspective. When the societal perspective is applied intervention costs (costs directly related to the delivery of the orthosis) healthcare costs (costs related to visits to general practitioners, medical specialists, and/or therapists, medication use and assistive devices) informal care costs, unpaid productivity costs, as well as costs related to productivity losses due to being absent from work (absenteeism) and productivity losses due to reduced productivity while being at work (presenteeism) will be included. When the healthcare

For peer review only

perspective is applied, only costs accruing to the formal Dutch healthcare system will be included. To collect data on resource use, participants are asked to fill in cost questionnaires at baseline (T1), directly after delivery of the orthosis and at 3 and 6 months post orthotic treatment (T2 and T3). All cost categories will be valued in accordance with the Dutch manual for costing studies in health care[40].

Outcome measures used for the economic evaluation will be Quality Adjusted Life Years (QALYs), walking energy cost and GAS scores. QALYs will be based on the EQ-5D-5L, administered at each measurement point (T1, T2 and T3). The patients' EQ-5D-5L health states will be converted into utility scores using the Dutch tariff[41]. Subsequently, QALYs will be estimated by multiplying the patients' utility scores by the time spent in a certain health state.

Statistical analysis

Walking energy cost and secondary outcomes at each measurement point (T1, T2 and T3) will be analyzed with linear mixed models for repeated measurements to investigate the effectiveness of specialized orthotic care compared to usual orthotic care over time, adjusted for stratification and differences in baseline scores. Time and study group will be included as dependent variables. The differences in GAS scores between groups at 3 and 6 months follow-up will be tested with non-parametric Mann-Whitney Wilcoxon tests, as GAS scores are ordinal. All comparisons between groups are based on an intention-to-treat analysis.

For the economic evaluation, mean differences in total costs and effects between groups will be estimated using seemingly unrelated regression analyses. To account for the skewed nature of cost data, 95% confidence intervals surrounding cost differences will be estimated using Bias Corrected and Accelerated bootstrapping with 5000 replication. Subsequently, incremental cost-effectiveness ratios will be calculated by dividing the mean differences in costs by the mean differences in effects. To illustrate the joint uncertainty surrounding costs and effects, cost-effectiveness planes and cost-effectiveness acceptability curves will be plotted. In a cost-effectiveness acceptability curve, the probability of specialized orthotic care being cost-effective compared to usual orthotic care is plotted for a range of willingness to pay values (i.e. the amount of money decision-makers are willing to pay per unit of effect gained).

All statistical analyses will be performed using SPSS (V25; IBM SPSS, Chicago, Illinois, USA) and a statistical significance of $p < 0.05$ will be used in this study.

Patient and public involvement

Patients were involved in the preparation and design of this study, will be kept informed about the progress during the conductance of the trial and will be involved in the reporting and dissemination of the results.

For peer review only

DISCUSSION

The aim of this study is to examine the effectiveness and cost-effectiveness of specialized orthotic care compared to usual orthotic care on functioning in adults with slowly progressive NMD. This study has several strengths.

First, a broad range of outcome measures on consecutive time points before and after orthotic treatment will be collected. For the examination of effectiveness, we will not only analyze objective outcome measures, such as walking energy cost and gait biomechanics, but also patient-reported outcome measures, like satisfaction with the provided orthosis. Besides, personal treatment goals will be set at the activity and participation level of the ICF and are therefore considered to be highly relevant to the patient. By assessing outcome measures from multiple perspectives, we will be able to fully capture the functional effects of specialized orthotic care and, at the same time, gain insight in the underlying biomechanical working mechanisms. Additionally, compared to previous research, evidence will be obtained in a large group of adults suffering from leg muscle weakness, caused by many different slowly progressive neuromuscular diseases, which increases the generalizability of results.

Second, participants in the control group will be treated in a diverse sample of usual care centers in which leg orthoses are prescribed throughout the Netherlands. This allows for a broad comparison between specialized orthotic care and orthotic care as applied in current practice, which could be of importance for the improvement of overall quality and efficiency of orthotic care. On the other hand, treatment components of the usual care process are expected to differ amongst centers due to local policies and available facilities. This could complicate the identification of underlying mechanisms of action that could explain differences in treatment outcomes between groups. To enable cautious exploration of potential mechanisms that might lead to beneficial treatment outcomes, which could be useful for future implications, the process of usual orthotic care will be extensively documented.

Finally, a major strength is the economic evaluation that will be performed alongside the study. Since expensive orthotic devices increasingly become available due to technological developments, it is important to not only assess whether orthotic devices are effective, but also whether their additional health effects are worth their additional costs. This study will be the first to provide insights into the cost-effectiveness of orthotic care in NMD in general and of specialized orthotic care compared to usual orthotic care in particular.

In conclusion, this study aims to examine the effectiveness and cost-effectiveness of specialized orthotic care compared to usual orthotic care in adults with NMD. Insights could lead to improvements in the quality of orthotic care resulting in improvements in overall functionality of adults with NMD in daily living. Consequently, insights could lead to a more efficient use of already scarce (healthcare) resources.

For peer review only

ETHICS AND DISSEMINATION

The study protocol was approved by the Medical Ethics committee of the Amsterdam UMC, location Academic Medical Center (AMC), ABR-number 67268. The study is registered at the Dutch Trial Register (NL7511) and will be performed in accordance with good clinical practice guidelines. An independent monitor of the Amsterdam UMC will monitor the study multiple times during the conduction of the study. Aspects that will be monitored will include inclusion rate, trial master file, informed consent process, inclusion and exclusion criteria, randomization, trial procedures, source data verification, safety reporting and closing and reporting. The investigator will be encouraged to maintain the blinding as far as possible and code breaks should only occur in exceptional circumstances. Important protocol changes will be recorded (a new protocol version number will be assigned) and reported to the Medical Ethics Committee. When patients sign their informed consent, they will receive a participant ID which will be coupled to all the data collected. Forms will be stored in a locked cabinet to assure anonymity. Only persons involved in the study have access to these forms before and after the study. Insurance has been taken out for participation of patients in the study. After completion of the study, positive as well as negative or inconclusive results will be submitted to a peer-reviewed journal and presented at national and international scientific conferences. Furthermore, results will be disseminated through other media aimed at a broader audience including patients. The study data sets and codes of the data analysis are available on request. Furthermore, participants will be informed about the results of the study by means of a newsletter.

AUTHOR STATEMENT

M-AB, SK and FN conceived the study. M-AB, FSK, FN, EvD and HvD contributed to the study design and methods. M-AB, FSK, FN, JT, RL, VA and EvD participated in logistical planning of the study. EvD wrote the manuscript and is responsible for data acquisition and analysis. All authors conceived, provided feedback and approved the final version of the manuscript.

FUNDING

This study was supported by ZonMw, The Netherlands Organisation for Health Research and Development, grant number: 853001103. The funder will have no role in the execution, analyses, interpretation of the data of the study, or decision to submit results.

COMPETING INTERESTS

None declared.

For peer review only

REFERENCES

1. Demir YP. Neuromuscular Diseases and Rehabilitation. In: Suzuki T. Neurological Physical Therapy. BoD – Books on Demand 2017.
2. van der Krogt MM, Delp SL, Schwartz MH. How robust is human gait to muscle weakness? *Gait & posture* 2012;36(1):113-9. doi: 10.1016/j.gaitpost.2012.01.017 [published Online First: 2012/03/06]
3. Brehm M, Nollet F, Harlaar J. Energy demands of walking in persons with postpoliomyelitis syndrome: relationship with muscle strength and reproducibility. *Arch Phys Med Rehabil* 2006;87(1):136-40. doi: 10.1016/j.apmr.2005.08.123
4. Menotti F, Felici F, Damiani A, et al. Charcot-Marie-Tooth 1A patients with low level of impairment have a higher energy cost of walking than healthy individuals. *Neuromuscul Disord* 2011;21(1):52-7. doi: 10.1016/j.nmd.2010.09.008 [published Online First: 2010/11/16]
5. Abresch RT, Carter GT, Jensen MP, et al. Assessment of pain and health-related quality of life in slowly progressive neuromuscular disease. *Am J Hosp Palliat Care* 2002;19(1):39-48. doi: 10.1177/104990910201900109 [published Online First: 2002/08/14]
6. Bickerstaffe A, Beelen A, Nollet F. Circumstances and consequences of falls in polio survivors. *J Rehabil Med* 2010;42(10):908-15. doi: 10.2340/16501977-0620 [published Online First: 2010/10/30]
7. Pieterse AJ, Luttikhold TB, de Laat K, et al. Falls in patients with neuromuscular disorders. *Journal of the neurological sciences* 2006;251(1-2):87-90. doi: 10.1016/j.jns.2006.09.008 [published Online First: 2006/11/14]
8. Ahlstrom G, Sjoden PO. Coping with illness-related problems and quality of life in adult individuals with muscular dystrophy. *J Psychosom Res* 1996;41(4):365-76. doi: 10.1016/s0022-3999(96)00191-2 [published Online First: 1996/10/01]
9. Johnson NE, Heatwole CR, Dilek N, et al. Quality-of-life in Charcot-Marie-Tooth disease: the patient's perspective. *Neuromuscul Disord* 2014;24(11):1018-23. doi: 10.1016/j.nmd.2014.06.433 [published Online First: 2014/08/06]
10. Hsu JD, Michael JW, Fisk JR. Atlas of orthoses and assistive devices. In: Surgeons AAoO, ed. 4th edition ed. Philadelphia: American Academy of Orthopedic Surgeons 2008:299-309.
11. ISO. ISO 8549-1 - Vocabulary - Part 1: general terms for external limb prostheses and external orthoses. Genève: International Organization for Standardization, 1989.
12. Hijmans JM, Geertzen JH. Development of clinical guidelines for the prescription of orthoses in patients with neurological disorders in The Netherlands. *Prosthet Orthot Int* 2006;30(1):35-43. doi: 10.1080/03093640500533232 [published Online First: 2006/06/03]
13. Heim S, Jacobs NA, Jensen JS, et al. ISPO consensus conference on poliomyelitis. Consensus Statement and Syndicate Reports. International Society of Prosthetics and Orthotics 1997.
14. van der Wilk D, Dijkstra PU, Postema K, et al. Effects of ankle foot orthoses on body functions and activities in people with floppy paretic ankle muscles: a systematic review. *Clin Biomech (Bristol, Avon)* 2015;30(10):1009-25. doi: 10.1016/j.clinbiomech.2015.09.013 [published Online First: 2015/11/21]
15. McDaid C, Fayter D, Booth A, et al. Systematic review of the evidence on orthotic devices for the management of knee instability related to neuromuscular and central nervous system disorders. *BMJ Open* 2017;7(9):e015927. doi: 10.1136/bmjopen-2017-015927 [published Online First: 2017/09/08]
16. Brehm MA, Beelen A, Doorenbosch CA, et al. Effect of carbon-composite knee-ankle-foot orthoses on walking efficiency and gait in former polio patients. *J Rehabil Med* 2007;39(8):651-7. doi: 10.2340/16501977-0110 [published Online First: 2007/09/27]

For peer review only

17. Waterval NFJ, Brehm MA, Harlaar J, et al. Description of orthotic properties and effects evaluation of ankle-foot orthoses in non-spastic calf muscle weakness. *J Rehabil Med* 2020 doi: 10.2340/16501977-2642 [published Online First: 2020/01/15]
18. Brehm M, Nollet F. Beenorthesen bij neuromusculaire aandoeningen. Amsterdam: Reed business education 2014.
19. College van Zorgverzekeringen. Procesbeschrijving hulpmiddelenzorg. Den Haag: Nationaal ICT instituut in de Zorg 2009.
20. WHO. International classification of functioning, disability and health: ICF. Genève: World Health Organization, 2001.
21. ISO. ISO 9999 Assistive products for persons with disability – Classification and terminology. Draft International Standard. Genève: International Organization for Standardization, 2010.
22. Hebert JS. Ambulatory KAFOs: A Physiatry Perspective. *J Prosthet Orthot* 2006;18(7 Proceedings):169-74.
23. Brehm M, Bus SA, Harlaar J, et al. A candidate core set of outcome measures based on the International Classification of Functioning, Disability and Health for clinical studies on lower limb orthoses. *Prosthet Orthot Int* 2011;35(3):269-77. doi: 10.1177/0309364611413496 [published Online First: 2011/09/23]
24. Waterval NFJ, Nollet F, Harlaar J, et al. Modifying ankle foot orthosis stiffness in patients with calf muscle weakness: gait responses on group and individual level. *J Neuroeng Rehabil* 2019;16(1):120. doi: 10.1186/s12984-019-0600-2 [published Online First: 2019/10/19]
25. Healy A, Farmer S, Eddison N, et al. A scoping literature review of studies assessing effectiveness and cost-effectiveness of prosthetic and orthotic interventions. *Disabil Rehabil Assist Technol* 2019:1-7. doi: 10.1080/17483107.2018.1523953 [published Online First: 2019/01/18]
26. Vinci P, Gargiulo P. Poor compliance with ankle-foot-orthoses in Charcot-Marie-Tooth disease. *Eur J Phys Rehabil Med* 2008;44(1):27-31. [published Online First: 2008/04/04]
27. Swinnen E, Kerckhofs E. Compliance of patients wearing an orthotic device or orthopedic shoes: A systematic review. *J Bodyw Mov Ther* 2015;19(4):759-70. doi: 10.1016/j.jbmt.2015.06.008 [published Online First: 2015/11/26]
28. Chan AW, Tetzlaff JM, Altman DG, et al. SPIRIT 2013 Statement: Defining standard protocol items for clinical trials. *Ann Intern Med* 2013;158(3):200-207. doi: 10.7326/0003-4819-158-3-201302050-00583 [published Online First: 2013/01/09]
29. Council MR. *Aids to examination of the peripheral nervous system*. Memorandum no 45. London: Her Majesty's Stationary Office 1976.
30. Garby L, Astrup A. The relationship between the respiratory quotient and the energy equivalent of oxygen during simultaneous glucose and lipid oxidation and lipogenesis. *Acta physiologica Scandinavica* 1987;129(3):443-4. doi: 10.1111/j.1365-201x.1987.tb10613.x [published Online First: 1987/03/01]
31. Brehm MA, Verduijn S, Bon J, et al. Comparison of two 6-minute walk tests to assess walking capacity in polio survivors. *J Rehabil Med* 2017;49(9):732-37. doi: 10.2340/16501977-2264 [published Online First: 2017/09/21]
32. Kempen JC, Harlaar J, van der Kooi AJ, et al. Reliability of the walking energy cost test and the six-minute walk test in boys with Duchenne muscular dystrophy. *Neuromuscul Disord* 2014;24(3):216-21. doi: 10.1016/j.nmd.2013.11.015 [published Online First: 2013/12/25]
33. Steenbeek D, Ketelaar M, Galama K, et al. Goal attainment scaling in paediatric rehabilitation: a critical review of the literature. *Dev Med Child Neurol* 2007;49(7):550-6. doi: 10.1111/j.1469-8749.2007.00550.x [published Online First: 2007/06/27]
34. Steenbeek D, Meester-Delver A, Becher JG, et al. The effect of botulinum toxin type A treatment of the lower extremity on the level of functional abilities in children with cerebral palsy: evaluation with goal attainment scaling. *Clin Rehabil* 2005;19(3):274-82. doi: 10.1191/0269215505cr859oa [published Online First: 2005/04/30]
35. Dekkers K, de Viet E, Eilander H, et al. Goal attainment scaling (GAS) in de praktijk [Goal attainment scaling in clinical practice], 2011.

For peer review only

36. Gandek B, Ware JE, Jr., Aaronson NK, et al. Tests of data quality, scaling assumptions, and reliability of the SF-36 in eleven countries: results from the IQOLA Project. International Quality of Life Assessment. *J Clin Epidemiol* 1998;51(11):1149-58. doi: 10.1016/s0895-4356(98)00106-1 [published Online First: 1998/11/17]
37. Kempen GI, Yardley L, van Haastregt JC, et al. The Short FES-I: a shortened version of the falls efficacy scale-international to assess fear of falling. *Age Ageing* 2008;37(1):45-50. doi: 10.1093/ageing/afm157 [published Online First: 2007/11/23]
38. Krupp LB, LaRocca NG, Muir-Nash J, et al. The fatigue severity scale. Application to patients with multiple sclerosis and systemic lupus erythematosus. *Arch Neurol* 1989;46(10):1121-3. doi: 10.1001/archneur.1989.00520460115022 [published Online First: 1989/10/01]
39. Wessels RD, De Witte LP. Reliability and validity of the Dutch version of QUEST 2.0 with users of various types of assistive devices. *Disabil Rehabil* 2003;25(6):267-72. doi: 10.1080/0963828021000031197 [published Online First: 2003/03/08]
40. Kanters TA, Bouwmans CAM, van der Linden N, et al. Update of the Dutch manual for costing studies in health care. *PloS one* 2017;12(11):e0187477. doi: 10.1371/journal.pone.0187477 [published Online First: 2017/11/10]
41. Versteegh MM, Vermeulen KM, Evers SM, et al. Dutch Tariff for the Five-Level Version of EQ-5D. *Value health* 2016;19(4):343-52. doi: 10.1016/j.jval.2016.01.003 [published Online First: 2016/06/22]

For peer review only

Figure legends

Figure 1: *Overview of the study design.*

For peer review only

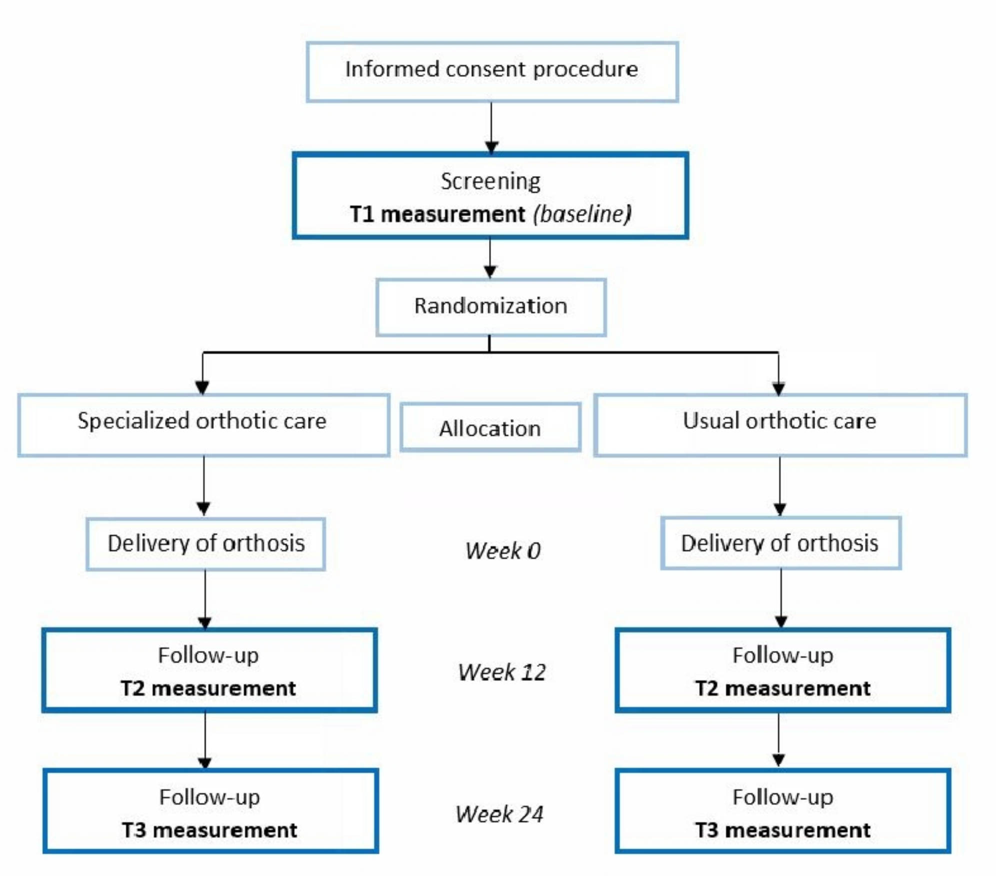

Figure 1: Overview of the study design.

209x182mm (96 x 96 DPI)

Reporting checklist for protocol of a clinical trial.

Based on the SPIRIT guidelines.

Instructions to authors

Complete this checklist by entering the page numbers from your manuscript where readers will find each of the items listed below.

Your article may not currently address all the items on the checklist. Please modify your text to include the missing information. If you are certain that an item does not apply, please write "n/a" and provide a short explanation.

Upload your completed checklist as an extra file when you submit to a journal.

In your methods section, say that you used the SPIRITreporting guidelines, and cite them as:

Chan A-W, Tetzlaff JM, Altman DG, Laupacis A, Gøtzsche PC, Krleža-Jerić K, Hróbjartsson A, Mann H, Dickersin K, Berlin J, Doré C, Parulekar W, Summerskill W, Groves T, Schulz K, Sox H, Rockhold FW, Rennie D, Moher D. SPIRIT 2013 Statement: Defining standard protocol items for clinical trials. Ann Intern Med. 2013;158(3):200-207

		Reporting Item	Page Number
**Administrative information**			
Title	#1	Descriptive title identifying the study design, population, interventions, and, if applicable, trial acronym	1
Trial registration	#2a	Trial identifier and registry name. If not yet registered, name of intended registry	2
Trial registration: data set	#2b	All items from the World Health Organization Trial Registration Data Set	1-12
Protocol version	#3	Date and version identifier	1
Funding	#4	Sources and types of financial, material, and other support	12
Roles and responsibilities: contributorship	#5a	Names, affiliations, and roles of protocol contributors	1, 12

For peer review only

Roles and responsibilities: sponsor contact information	#5b	Name and contact information for the trial sponsor	1
Roles and responsibilities: sponsor and funder	#5c	Role of study sponsor and funders, if any, in study design; collection, management, analysis, and interpretation of data; writing of the report; and the decision to submit the report for publication, including whether they will have ultimate authority over any of these activities	12
Roles and responsibilities: committees	#5d	Composition, roles, and responsibilities of the coordinating centre, steering committee, endpoint adjudication committee, data management team, and other individuals or groups overseeing the trial, if applicable (see Item 21a for data monitoring committee)	12
**Introduction**			
Background and rationale	#6a	Description of research question and justification for undertaking the trial, including summary of relevant studies (published and unpublished) examining benefits and harms for each intervention	4
Background and rationale: choice of comparators	#6b	Explanation for choice of comparators	5
Objectives	#7	Specific objectives or hypotheses	5
Trial design	#8	Description of trial design including type of trial (eg, parallel group, crossover, factorial, single group), allocation ratio, and framework (eg, superiority, equivalence, non-inferiority, exploratory)	6
**Methods: Participants, interventions, and outcomes**			
Study setting	#9	Description of study settings (eg, community clinic, academic hospital) and list of countries where data will be collected. Reference to where list of study sites can be	6

For peer review only

		obtained	
Eligibility criteria	#10	Inclusion and exclusion criteria for participants. If applicable, eligibility criteria for study centres and individuals who will perform the interventions (eg, surgeons, psychotherapists)	6
Interventions: description	#11a	Interventions for each group with sufficient detail to allow replication, including how and when they will be administered	7
Interventions: modifications	#11b	Criteria for discontinuing or modifying allocated interventions for a given trial participant (eg, drug dose change in response to harms, participant request, or improving / worsening disease)	n/a
Interventions: adherance	#11c	Strategies to improve adherence to intervention protocols, and any procedures for monitoring adherence (eg, drug tablet return; laboratory tests)	n/a
Interventions: concomitant care	#11d	Relevant concomitant care and interventions that are permitted or prohibited during the trial	n/a
Outcomes	#12	Primary, secondary, and other outcomes, including the specific measurement variable (eg, systolic blood pressure), analysis metric (eg, change from baseline, final value, time to event), method of aggregation (eg, median, proportion), and time point for each outcome. Explanation of the clinical relevance of chosen efficacy and harm outcomes is strongly recommended	8
Participant timeline	#13	Time schedule of enrolment, interventions (including any run-ins and washouts), assessments, and visits for participants. A schematic diagram is highly recommended (see Figure)	6
Sample size	#14	Estimated number of participants needed to achieve study objectives and how it was determined, including clinical and statistical assumptions supporting any sample size calculations	6
Recruitment	#15	Strategies for achieving adequate participant enrolment to reach target sample size	6

For peer review only

**Methods: Assignment of interventions (for controlled trials)**			
Allocation: sequence generation	#16a	Method of generating the allocation sequence (eg, computer-generated random numbers), and list of any factors for stratification. To reduce predictability of a random sequence, details of any planned restriction (eg, blocking) should be provided in a separate document that is unavailable to those who enrol participants or assign interventions	6-7
Allocation concealment mechanism	#16b	Mechanism of implementing the allocation sequence (eg, central telephone; sequentially numbered, opaque, sealed envelopes), describing any steps to conceal the sequence until interventions are assigned	6-7
Allocation: implementation	#16c	Who will generate the allocation sequence, who will enrol participants, and who will assign participants to interventions	6
Blinding (masking)	#17a	Who will be blinded after assignment to interventions (eg, trial participants, care providers, outcome assessors, data analysts), and how	7
Blinding (masking): emergency unblinding	#17b	If blinded, circumstances under which unblinding is permissible, and procedure for revealing a participant's allocated intervention during the trial	12
**Methods: Data collection, management, and analysis**			
Data collection plan	#18a	Plans for assessment and collection of outcome, baseline, and other trial data, including any related processes to promote data quality (eg, duplicate measurements, training of assessors) and a description of study instruments (eg, questionnaires, laboratory tests) along with their reliability and validity, if known. Reference to where data collection forms can be found, if not in the	8-10

For peer review only

		protocol	
Data collection plan: retention	#18b	Plans to promote participant retention and complete follow-up, including list of any outcome data to be collected for participants who discontinue or deviate from intervention protocols	12
Data management	#19	Plans for data entry, coding, security, and storage, including any related processes to promote data quality (eg, double data entry; range checks for data values). Reference to where details of data management procedures can be found, if not in the protocol	12
Statistics: outcomes	#20a	Statistical methods for analysing primary and secondary outcomes. Reference to where other details of the statistical analysis plan can be found, if not in the protocol	10
Statistics: additional analyses	#20b	Methods for any additional analyses (eg, subgroup and adjusted analyses)	10
Statistics: analysis population and missing data	#20c	Definition of analysis population relating to protocol non-adherence (eg, as randomised analysis), and any statistical methods to handle missing data (eg, multiple imputation)	10
**Methods: Monitoring**			
Data monitoring: formal committee	#21a	Composition of data monitoring committee (DMC); summary of its role and reporting structure; statement of whether it is independent from the sponsor and competing interests; and reference to where further details about its charter can be found, if not in the protocol. Alternatively, an explanation of why a DMC is not needed	12
Data monitoring: interim analysis	#21b	Description of any interim analyses and stopping guidelines, including who will have access to these interim results and make the final decision to terminate the trial	12
Harms	#22	Plans for collecting, assessing, reporting, and managing solicited and spontaneously reported adverse events and other unintended effects of trial interventions or trial conduct	6
Auditing	#23	Frequency and procedures for auditing trial conduct, if	12

For peer review only

		any, and whether the process will be independent from investigators and the sponsor	
**Ethics and dissemination**			
Research ethics approval	#24	Plans for seeking research ethics committee / institutional review board (REC / IRB) approval	12
Protocol amendments	#25	Plans for communicating important protocol modifications (eg, changes to eligibility criteria, outcomes, analyses) to relevant parties (eg, investigators, REC / IRBs, trial participants, trial registries, journals, regulators)	12
Consent or assent	#26a	Who will obtain informed consent or assent from potential trial participants or authorised surrogates, and how (see Item 32)	6
Consent or assent: ancillary studies	#26b	Additional consent provisions for collection and use of participant data and biological specimens in ancillary studies, if applicable	n/a
Confidentiality	#27	How personal information about potential and enrolled participants will be collected, shared, and maintained in order to protect confidentiality before, during, and after the trial	12
Declaration of interests	#28	Financial and other competing interests for principal investigators for the overall trial and each study site	12
Data access	#29	Statement of who will have access to the final trial dataset, and disclosure of contractual agreements that limit such access for investigators	12
Ancillary and post trial care	#30	Provisions, if any, for ancillary and post-trial care, and for compensation to those who suffer harm from trial participation	12
Dissemination policy: trial results	#31a	Plans for investigators and sponsor to communicate trial results to participants, healthcare professionals, the public, and other relevant groups (eg, via publication, reporting in results databases, or other data sharing arrangements), including any publication restrictions	12
Dissemination policy:	#31b	Authorship eligibility guidelines and any intended use of	12

For peer review only

authorship		professional writers	
Dissemination policy: reproducible research	#31c	Plans, if any, for granting public access to the full protocol, participant-level dataset, and statistical code	12
**Appendices**			
Informed consent materials	#32	Model consent form and other related documentation given to participants and authorised surrogates	n/a
Biological specimens	#33	Plans for collection, laboratory evaluation, and storage of biological specimens for genetic or molecular analysis in the current trial and for future use in ancillary studies, if applicable	n/a

The SPIRIT checklist is distributed under the terms of the Creative Commons Attribution License CC-BY-ND 3.0. This checklist was completed on 22. April 2020 using https://www.goodreports.org/, a tool made by the EQUATOR Network in collaboration with Penelope.ai

For peer review only

BMJ Open

Specialized orthotic care to improve functioning in adults with neuromuscular disorders: protocol of a prospective randomized open label blinded end-point study.

Journal:	*BMJ Open*
Manuscript ID	bmjopen-2020-039683.R1
Article Type:	Protocol
Date Submitted by the Author:	03-Aug-2020
Complete List of Authors:	van Duijnhoven, Elza; Amsterdam UMC, University of Amsterdam, Department of Rehabilitation Medicine, Amsterdam Movement Sciences<br>Koopman, Fieke; Amsterdam UMC, University of Amsterdam, Department of Rehabilitation Medicine, Amsterdam Movement Sciences<br>Tuijtelaars, Jana; Amsterdam UMC, University of Amsterdam, Department of Rehabilitation Medicine, Amsterdam Movement Sciences<br>Altmann, Viola; Klimmendaal, Rehabilitation center<br>Lagrand, Rimke; Amsterdam UMC, University of Amsterdam, Department of Rehabilitation Medicine, Amsterdam Movement Sciences<br>van Dongen, Johanna; Vrije Universiteit Amsterdam, Department of Health Sciences, Faculty of Science<br>Nollet, Frans; Amsterdam UMC, University of Amsterdam, Department of Rehabilitation Medicine, Amsterdam Movement Sciences<br>Brehm, Merel-Anne; Amsterdam UMC, University of Amsterdam, Department of Rehabilitation Medicine, Amsterdam Movement Sciences
**Primary Subject Heading**:	Rehabilitation medicine
Secondary Subject Heading:	Neurology
Keywords:	Neuromuscular disease < NEUROLOGY, REHABILITATION MEDICINE, Protocols & guidelines < HEALTH SERVICES ADMINISTRATION & MANAGEMENT

SCHOLARONE™ Manuscripts

I, the Submitting Author has the right to grant and does grant on behalf of all authors of the Work (as defined in the below author licence), an exclusive licence and/or a non-exclusive licence for contributions from authors who are: i) UK Crown employees; ii) where BMJ has agreed a CC-BY licence shall apply, and/or iii) in accordance with the terms applicable for US Federal Government officers or employees acting as part of their official duties; on a worldwide, perpetual, irrevocable, royalty-free basis to BMJ Publishing Group Ltd ("BMJ") its licensees and where the relevant Journal is co-owned by BMJ to the co-owners of the Journal, to publish the Work in this journal and any other BMJ products and to exploit all rights, as set out in our licence.

The Submitting Author accepts and understands that any supply made under these terms is made by BMJ to the Submitting Author unless you are acting as an employee on behalf of your employer or a postgraduate student of an affiliated institution which is paying any applicable article publishing charge ("APC") for Open Access articles. Where the Submitting Author wishes to make the Work available on an Open Access basis (and intends to pay the relevant APC), the terms of reuse of such Open Access shall be governed by a Creative Commons licence – details of these licences and which Creative Commons licence will apply to this Work are set out in our licence referred to above.

Other than as permitted in any relevant BMJ Author's Self Archiving Policies, I confirm this Work has not been accepted for publication elsewhere, is not being considered for publication elsewhere and does not duplicate material already published. I confirm all authors consent to publication of this Work and authorise the granting of this licence.

For peer review only

Specialized orthotic care to improve functioning in adults with neuromuscular disorders: protocol of a prospective randomized open label blinded end-point study.

Elza van Duijnhoven[1], Fieke Sophia Koopman[1], Jana Tuijtelaars[1], Viola Altmann[2], Rimke Lagrand[1], Johanna Maria van Dongen[3], Frans Nollet[1], Merel-Anne Brehm[1]

[1]Amsterdam UMC, University of Amsterdam, Department of Rehabilitation Medicine, Amsterdam Movement Sciences, Meibergdreef 9, Amsterdam, the Netherlands

[2]Klimmendaal, Rehabilitation center, Arnhem, the Netherlands.

[3]Department of Health Sciences, Faculty of Science, Vrije Universiteit Amsterdam ,Amsterdam Movement Sciences, Amsterdam, the Netherlands

*Correspondence to: Elza van Duijnhoven

Department of Rehabilitation Medicine

Amsterdam UMC, location Academic Medical Center

Meibergdreef 9

1105 AZ, Amsterdam

The Netherlands

Email: e.vanduijnhoven@amsterdamumc.nl

Telephone: +31 20 566 6915

Fax: +31 20 5669154

Issue date: 28-07-2020

Word count: 4000

For peer review only - http://bmjopen.bmj.com/site/about/guidelines.xhtml

ABSTRACT

Introduction

People suffering from leg muscle weakness caused by neuromuscular disorders (NMD) are often provided with leg orthoses to reduce walking problems such as increased walking effort, diminished walking speed, reduced balance and falls. However, evidence for the effectiveness of leg orthoses to improve walking in this patient group is limited and there is an absence of standardized practice in orthotic prescription. In 2012 a Dutch multidisciplinary guideline was developed aimed to standardize the orthotic treatment process in NMD. Although application of the guideline in expert centers (specialized orthotic care) seems beneficial regarding clinical effectiveness, larger studies are necessary to confirm results and investigate cost-effectiveness. Therefore, this study aims to examine the effectiveness and cost-effectiveness of specialized orthotic care compared to usual orthotic care in adults with slowly progressive NMD.

Methods and analysis

A prospective randomized open label blinded end-point study will be performed, in which 70 adults with slowly progressive NMD are randomly assigned to specialized orthotic care (intervention) or usual orthotic care (control). Outcome measures are assessed at baseline and at 3 and 6 months follow-up. The primary endpoints are gross walking energy cost (J/kg/m) assessed during a 6-minute walk test and achievement of personal goals, measured with the goal attainment scale. Secondary endpoints include walking speed, gait biomechanics, stability, physical functioning, falls and fear of falling, perceived fatigue, and satisfaction. For the economic evaluation, societal costs and health-related quality of life will be assessed using cost questionnaires and the EQ-5D-5L, retrospectively.

Ethics and dissemination

The study is registered in the Dutch trial register (NL 7511) and the protocol has been approved by the Medical Ethics Committee of the Academic Medical Center in Amsterdam. Results will be presented at national and international scientific conferences and disseminated through peer-reviewed journals and media aimed at a broad audience including patients.

Keywords

Neuromuscular diseases; orthotic devices; specialized orthotic care; cost-effectiveness, walking energy cost, goal attainment scale; RCT.

For peer review only

For peer review only - http://bmjopen.bmj.com/site/about/guidelines.xhtml

ARTICLE SUMMARY

Strengths and limitations of this study

- A broad range of outcome measures, both objective and patient-reported, will be collected before and after orthotic treatment, resulting in a unique dataset of both functional effects, underlying biomechanical working mechanisms, and costs.
- An economic evaluation will be performed alongside the study to provide insights into the cost-effectiveness of specialized orthotic care versus usual orthotic care.
- A possible limitation is the expected variability in treatment components of usual orthotic care, which could complicate the identification of underlying mechanisms of action between treatments. The process of usual orthotic care will therefore be extensively documented.

For peer review only

For peer review only - http://bmjopen.bmj.com/site/about/guidelines.xhtml

INTRODUCTION

People with slowly progressive neuromuscular disorders (NMD), such as post-polio syndrome and Charcot Marie Tooth disease, frequently suffer from leg muscle weakness[1]. Leg muscle weakness will change the gait pattern[2], causing walking problems such as increased walking effort[3 4], diminished walking speed[3 4], pain[5], balance problems and falls[6 7]. These walking problems may restrict the patients' performance of daily physical activities[8] and negatively affect their independency and quality of life[8 9]. Due to the progressive nature of NMD, walking problems will gradually increase over time[9], which will further negatively affect physical mobility, independency and quality of life.

Leg orthoses are provided to reduce walking problems in people with NMD[10]. A leg orthosis is an "externally applied medical device encompassing (part of) the leg and foot, used to modify the structural and functional characteristics of the neuromuscular and skeletal systems"[11]. In general, an ankle-foot orthosis (AFO) is provided in case of distal leg muscle weakness, which often includes weakness of the foot dorsiflexors and plantarflexors[10 12]. Accordingly, the AFO should support foot clearance during swing by restricting plantarflexion and compensate for plantarflexor weakness by restricting dorsiflexion during late stance[10]. Knee-ankle-foot orthoses (KAFOs) are provided for (additional) proximal leg muscle weakness, particularly weakness of the quadriceps, to stabilize the knee joint during the stance phase of gait and allow safe weight-bearing[10 13].

In previous research, various types of AFOs for calf muscle weakness[14] and KAFOs for quadriceps weakness have been found to improve walking in people with NMD[15]. However, the strength of the evidence is rather limited due to a lack of proper study designs, relatively small sample sizes, and heterogeneity in types of leg orthoses and control conditions studied. Accordingly, there is a lack of evidence-based and standardized guidance on the application of leg orthoses. Furthermore, many different types of leg orthoses are being prescribed in current orthotic practice in NMD, varying largely in orthotic properties and effectiveness, with both good and suboptimal treatment outcomes in terms of improving walking[16 17].

In 2012, a Dutch multidisciplinary guideline for leg orthoses in adults with slowly progressive NMD was developed with the aim to standardize the treatment process and improve treatment outcomes[18]. The guideline was developed according to proposed national and international frameworks[19 20 21] and compromises the entire process of orthotic treatment in systematically divided steps, as well as treatment algorithms for the selection of leg orthoses. According to the guideline, the care need should be individually characterized in terms of the personal health problems, goals and gait deviations to be addressed, caused by the underlying impairments. Based on the care need, the orthotic goals should be defined and matched with the orthosis design. Gait training after delivery of the orthosis after is essential to maximize effectiveness[22]. Finally, it is important to systematically evaluate the effectiveness as well as user experiences to ensure the functionality of the orthosis[23].

At this moment, the guideline is not widely applied throughout the Netherlands, as it requires sufficient expertise of the multidisciplinary care team involved and facilities for advanced 3D-gait analysis and gait training. However, prescribing leg orthoses according to the guideline (i.e. specialized orthotic care) seems promising in terms of clinical effectiveness as recently shown in two uncontrolled trials[16 24]. In individuals with calf muscle weakness due to NMD, individually stiffness-optimized AFOs have been shown to reduce walking effort to a much greater extent compared to AFOs prescribed in usual orthotic care[24]. Furthermore, in polio survivors[16], the increment in walking effort with KAFOs

For peer review only - http://bmjopen.bmj.com/site/about/guidelines.xhtml

prescribed in specialized orthotic care was reduced with 18% towards normative values, when compared to usual care KAFOs. At this moment, the effectiveness of leg orthoses prescribed within specialized orthotic care on functioning needs to be investigated in a larger group of individuals with slowly progressive NMD in comparison to usual orthotic care to strengthen evidence for the possible benefit.

While good-quality studies are needed to strengthen the evidence for the effectiveness of specialized orthotic care in NMD, it is also imperative to investigate whether the treatment is cost-effective, which is currently unknown. Since new and expensive orthotic devices increasingly become available, it is important to assess their associated costs[25]. Also, at this moment, an important problem in usual orthotic care concerns the low compliance of patients wearing their devices[26], which in turn leads to an inefficient use of healthcare resources[27]. Optimally matching orthoses with the personal needs and goals of the patient, according to the guideline, could lead to gains in clinical effectiveness and subsequently a higher compliance. In this respect, specialized orthotic care could improve the efficiency of (healthcare) resource, but this has not yet been investigated.

The aim of this study is to examine the effectiveness and cost-effectiveness of specialized orthotic care compared to usual orthotic care on functioning in adults with NMD. We hypothesize that specialized orthotic care is more effective in terms of reducing walking effort and achieving personal goals when compared to usual orthotic care. Furthermore, specialized orthotic care is expected to be cost-effective compared with usual orthotic care from a societal and healthcare perspective.

For peer review only

For peer review only - http://bmjopen.bmj.com/site/about/guidelines.xhtml

METHODS AND ANALYSIS

Study design

The study is designed as a randomized open label blinded end-point study that is prospectively registered at the Dutch Trial Register under number NL7511. The trial protocol was developed according to the SPIRIT guidelines[28] (supplementary file 1). Recruitment started in March 2019 and is foreseen to end at December 2021. Measurements are conducted at baseline (T1) and 3 and 6 months after orthotic treatment is given (T2 and T3, respectively). To determine the cost-effectiveness, an economic evaluation is performed alongside the study. An overview of the study design is shown in Figure 1.

Study population

We intend to include 70 adults with slowly progressive NMD (such as Charcot-Marie-Tooth disease, post-polio syndrome, inclusion body myositis or myotonic dystrophy) or peripheral nerve injury who have non-spastic leg muscle weakness. For eligibility, patients may or may not use an orthosis provided in usual care at the time of recruitment. Patients will be recruited from rehabilitation centers and hospitals throughout the Netherlands and through the Dutch Association for Neuromuscular Diseases. If a patient is willing to participate, a screening visit is planned. During the screening visit, written informed consent (supplementary file 2) is obtained and the investigator, in close collaboration with a rehabilitation physician, will check the in- and exclusion criteria.

Inclusion criteria are: (1) minimum age of 18 years, (2) weakness of the calf muscles (i.e. Medical Research Council (MRC) scale[29] score < 5 or not being able to make a heel-rise on one leg > 3 times) and/or weakness of the quadriceps (i.e. MRC score < 5) (3) experiencing walking problems such as increased walking effort, pain and/or impaired balance during standing and/or walking, (4) able to walk for 6 minutes at comfortable speed with or without assistive device (e.g. cane, crutch, walker), (5) indicated for an orthosis based upon physical examination and 3D gait analysis, and (6) motivated to use an orthosis. An exclusion criterion is: (1) insufficient mastery of the Dutch language.

Sample size

The sample size of this study is based on a power analysis of the expected differences in walking effort (defined as gross walking energy cost) and the achievement of personal treatment goals between the intervention and control group. According to previous studies[16 17], a difference in change in walking energy cost of 0.60 J/kg/m from baseline to 6 months post-treatment is expected between usual orthotic care and specialized orthotic care, where a difference of at least 0.45 J/kg/m is considered as clinical relevant[3]. Based on an intention-to-treat analysis, alpha of 0.05, power of 80%, and using an estimated correlation coefficient of the repeated measures of 0.76 and standard deviation of 0.90 J/kg/m, 30 patients per treatment group are necessary. The goal is to include 70 patients allowing for a 15% drop out. This sample size also allows the detection of 1 point difference in personal achievement goal scores (Based on an intention-to-treat analysis, alpha of 0.05, power of 80%, and using an estimated correlation coefficient of the repeated measures of 0.70).

Randomization and blinding

After baseline measurement (T1), participants will be randomly assigned in a 1:1 ratio to receive specialized orthotic care (intervention group) or usual orthotic care (control group). The randomization

For peer review only - http://bmjopen.bmj.com/site/about/guidelines.xhtml

scheme will be computer-generated in Castor (Castor EDC, Amsterdam, the Netherlands) and uses blocks of random sequences with variable sizes (2, 4 and 6). Patients will be stratified by disease severity defined as distal leg weakness versus (distal and) proximal leg weakness, respectively. Outcomes will be assessed by a blinded and independent assessor. When patients are informed of group allocation, they will be instructed not to reveal this to the outcome assessor.

Intervention

Specialized orthotic care

Patients in the intervention group will receive specialized orthotic care at an expert center that has implemented the Dutch guideline for leg orthoses[18] and has facilities for 3D gait analysis, gait training and fabricating custom-made leg orthoses. For the orthotic care process, protocols of the guideline will be used and treatment integrity checks will be performed to ensure compliance with the guideline.

First, the health problems and goals of the patient will assessed by means of an interview, questionnaires, physical examination and 3D gait analysis. Based on these assessments, the multidisciplinary care team will identify how the patients' problems and goals are best matched to an orthotic solution. The desired level of functioning for the patient and the required orthotic functions will be described in a care plan. Accordingly, the orthosis will be custom fabricated by an orthotic technician. After delivery of the orthosis, instructions on use and maintenance will be given to the patient and, when indicated, the patient receives gait training supervised by an experienced physiotherapist. Finally, after 3 months of using the orthosis, experiences of the patient as well as the desired level of functioning and orthotic functions will be evaluated. When needed, the orthosis will be adjusted based on the evaluation. The provision process will be extensively documented for each participant individually by an investigator who is not blinded.

Usual orthotic care

Participants in the control group will receive usual orthotic care from a rehabilitation physician at their regular care center. Treatment can vary across centers and may include provision of orthopedic shoes, provision of off-the-shelf or custom-made AFOs or provision of off-the-shelf or custom-made KAFOs according to the discretion of the practitioners and local policy of the center. For participants who already use an orthosis at baseline, usual orthotic care will concern the provision of a new orthosis which may be identical of differ from the orthosis being used. The provision process in the usual care centers will be extensively documented for each participant individually by an investigator who is not blinded.

Outcome measures

Relevant demographic variables, anthropometrics and clinical characteristics will be measured at baseline (T1). Clinical characteristics to be evaluated include; 1) manually assessed muscle strength of the left and right hip flexors and extensors, hip abductors and adductors, knee flexors and extensors, ankle plantar and dorsal flexors and ankle invertors and evertors, scored according to the MRC scale, and 2) passive range of motion of the hip, knee and ankle joints (left and right). Furthermore, 3) occurrence of joint deformities and 4) impairments in sensory function will be evaluated.

Primary outcomes and secondary outcomes (described below) will be assessed at baseline (T1) and at 3 and 6 months after orthotic treatment (T2 and T3, respectively). Additionally, orthotic mechanical

For peer review only - http://bmjopen.bmj.com/site/about/guidelines.xhtml

properties (such as type, weight, material and orthotic functions) and adverse events due to the use of the device (such as pressure sores, pain, muscle soreness) will be documented. The use of any medication during the study will be monitored at all measurement time points and documented in the cost questionnaires. All collected outcomes will be entered into a Castor database. An overview of all outcomes per measurement visit is given in Table 1.

***Table 1**: Overview of outcomes per measurement session.*

		Baseline	Follow-up	
		**T1 measurement** *screening*	**T2 measurement** *(12 weeks after delivery)*	**T3 measurement** *(24 weeks after delivery)*
**Primary outcomes**				
Walking energy cost	*6MWT*	X	X	X
Personal goals	*GAS*	Setting goals	X	X
**Secondary outcomes**				
Walking speed	*6MWT*	X	X	X
Gait biomechanics	*3DGA*	X	X	X
Stability	*NRS*	X	X	X
Physical functioning	*SF36-PF*	X	X	X
Fear of falling	*FES*	X	X	X
Fall rate	*Questionnaire*	X	X	X
Fatigue	*FSS*	X	X	X
Satisfaction	*D-Quest*	X*	X	X
**Additional outcomes**				
Demographics	*Intake*	X		
Anthropometrics	*Physical exam*	X		
Muscle strength	*Physical exam*	X		
Joint passive range of motion	*Physical exam*	X		
Sensory function	*Physical exam*	X		
Orthotic properties	*CRF*	X*	X	X
Adverse events	*CRF*		X	X
**Economic evaluation**				
Resource use**	*Cost questionnaire*	X	X	X
Health-related quality of life**	*EQ-5D-5L*	X	X	X

*Outcomes will only be assessed in case a participant uses an orthosis at baseline.
**Outcomes for the economic evaluation will also be assessed directly after delivery of the orthosis.

6MWT = 6-minute walk test, GAS = Goal Attainment Scale, 3DGA = 3-dimensional gait analysis, NRS = numeric rating scale, SF36-PF = physical functioning scale of the SF36, FES = Falls efficacy scale, FSS = Fatigue severity scale, D-Quest = Dutch version of the Quebec User Evaluation of Satisfaction with Assistive Technology, CRF = clinical report form, EQ-5D-5L = 5-level version of EuroQol 5D.

Primary outcomes

Primary outcomes are walking energy cost and the achievement of personal treatment goals. Walking energy cost will be determined during a 6-minute walk test (6MWT) in which participants walk at a self-selected comfortable speed on an indoor oval track. Simultaneously, oxygen uptake (VO_2) and the respiratory exchange ratio (RER) will be measured breath-by-breath with the Cosmed K5 portable gas analysis system (Cosmed, Rome, Italy). Mean VO_2, RER and walking speed values will be obtained at

For peer review only - http://bmjopen.bmj.com/site/about/guidelines.xhtml

steady state from the last 3 minutes of the 6MWT using a custom-written Matlab script (V.2019; MathWorks, Natick, Massachusetts, USA). From these outcomes, walking energy cost in J/kg/m will be calculated by using the following formula: ((4.940 x RER) + 16.040) x VO_2)/ walking speed where VO_2 is in ml/kg/min[30]. The assessment of walking energy cost has previously been shown to be reliable in patients with NMD[31 32].

The achievement of personal treatment goals will be quantified with the Goal Attainment Scale (GAS) to capture the diversity of orthotic treatment goals important to the individual patient[33]. At baseline, two goals that are highly relevant to the individual will be determined with the patient based on what they wish to achieve in daily life in terms of activities and participation according to the International Classification of Functioning, Disability and Health (ICF) framework[20]. Subsequently, personal GAS scales will be determined by creating six distinct levels of outcome ranging from -3 to +2, where the desired attainment goal of the patient is defined as 0 and the current situation is defined as -2. Achievement of the goals will be scored at T2 and T3 as -3 = worsened, -2 = unchanged, -1 = somewhat less than expected, 0 = expected outcome, +1 = somewhat more than expected and +2 = much more than expected. Improvements of at least 2 points will be regarded as clinical relevant[34]. The investigator defining and scoring the GAS, followed a course in applying GAS within a rehabilitation context[35].

Secondary outcomes

Secondary outcomes include walking speed (measured during the 6MWT), gait biomechanics (explained below), perceived stability during walking (assessed with a 11-point numeric rating scale (NRS)), perceived physical functioning (assessed with the Short-Form Health Survey physical functioning scale (SF36-PF)[36]), frequency of falls and fear of falling (assessed with the short version of the Falls Efficacy Scale (FES)[37], perceived fatigue (assessed with the Fatigue Severity Scale (FSS)[38]) and satisfaction with the orthosis (assessed with the Dutch version of the Quebec User Evaluation of Satisfaction with Assistive Technology (D-QUEST)[39] added with self-designed items).

Gait biomechanics

Gait biomechanics will be assessed with a 100 Hz 12-camera 3D motion capture system (VICON MX 1.3) and two adjacent 1000 Hz force plates (OR6-7; AMTI, Watertown, Marssachusetts, USA). Preparations include placement of reflective markers on the body according to the Plug-In Gait model. After a static calibration, participants will be asked to walk along a 12-m long walkway. Conditions that will be assessed include barefoot walking (T1), walking with shoes (T1), walking with the current orthosis if applicable (T1) and walking with the new orthosis (T2 and T3). Per condition, at least three valid trials will be used for analysis containing a clear stance phase on the force plate for both feet and full visibility of all markers during the gait cycle. Joint angles, net joint moments and joint powers around the hip, knee and ankle will be calculated per trial and averaged over three trials. Spatiotemporal gait parameters (such as step length and step width) and relevant kinematic and kinetic variables (such as maximal ankle dorsiflexion angle, peak ankle power, knee angle and knee moment at midstance, hip flexion angle and maximal hip, knee and ankle angle during swing and progression of the center of pressure) will be obtained from these averaged data and used for analysis.

Economic evaluation

For peer review only - http://bmjopen.bmj.com/site/about/guidelines.xhtml

The economic evaluation will be performed from a societal and a healthcare perspective. When the societal perspective is applied intervention costs (costs directly related to the delivery of the orthosis) healthcare costs (costs related to visits to general practitioners, medical specialists, and/or therapists, medication use and assistive devices) informal care costs, unpaid productivity costs, as well as costs related to productivity losses due to being absent from work (absenteeism) and productivity losses due to reduced productivity while being at work (presenteeism) will be included and assessed with a cost questionnaire. When the healthcare perspective is applied, only costs accruing to the formal Dutch healthcare system will be included. To collect data on resource use, participants are asked to fill in cost questionnaires at baseline (T1), directly after delivery of the orthosis and at 3 and 6 months post orthotic treatment (T2 and T3). All cost categories will be valued in accordance with the Dutch manual for costing studies in health care[40].

Outcome measures used for the economic evaluation will be Quality Adjusted Life Years (QALYs), walking energy cost and GAS scores. QALYs will be based on the EQ-5D-5L, administered at each measurement point (T1, T2 and T3). The patients' EQ-5D-5L health states will be converted into utility scores using the Dutch tariff[41]. Subsequently, QALYs will be estimated by multiplying the patients' utility scores by the time spent in a certain health state.

Statistical analysis

Walking energy cost and secondary outcomes at each measurement point (T1, T2 and T3) will be analyzed with linear mixed models for repeated measurements to investigate the effectiveness of specialized orthotic care compared to usual orthotic care over time, adjusted for stratification and differences in baseline scores. Time and study group will be included as dependent variables. The differences in GAS scores between groups at 3 and 6 months follow-up will be tested with non-parametric Mann-Whitney Wilcoxon tests, as GAS scores are ordinal. All comparisons between groups are based on an intention-to-treat analysis.

For the economic evaluation, mean differences in total costs and effects between groups will be estimated using seemingly unrelated regression analyses. To account for the skewed nature of cost data, 95% confidence intervals surrounding cost differences will be estimated using Bias Corrected and Accelerated bootstrapping with 5000 replication. Subsequently, incremental cost-effectiveness ratios will be calculated by dividing the mean differences in costs by the mean differences in effects. To illustrate the joint uncertainty surrounding costs and effects, cost-effectiveness planes and cost-effectiveness acceptability curves will be plotted. In a cost-effectiveness acceptability curve, the probability of specialized orthotic care being cost-effective compared to usual orthotic care is plotted for a range of willingness to pay values (i.e. the amount of money decision-makers are willing to pay per unit of effect gained).

All statistical analyses will be performed using SPSS (V25; IBM SPSS, Chicago, Illinois, USA) and a statistical significance of $p < 0.05$ will be used in this study.

Patient and public involvement

Patients were actively involved in the preparation of this study through participating in meetings with the research group in the development stage of the study protocol and by providing feedback on the study procedures and the patient information documentation. During the conductance of the study,

For peer review only - http://bmjopen.bmj.com/site/about/guidelines.xhtml

patients will be informed about the progress and involved in patient recruitment and the interpretation, reporting and dissemination of the results.

For peer review only

For peer review only - http://bmjopen.bmj.com/site/about/guidelines.xhtml

DISCUSSION

The aim of this study is to examine the effectiveness and cost-effectiveness of specialized orthotic care compared to usual orthotic care on functioning in adults with slowly progressive NMD. This study has several strengths.

First, a broad range of outcome measures on consecutive time points before and after orthotic treatment will be collected. For the examination of effectiveness, we will not only analyze objective outcome measures, such as walking energy cost and gait biomechanics, but also patient-reported outcome measures, like satisfaction with the provided orthosis. Besides, personal treatment goals will be set at the activity and participation level of the ICF and are therefore considered to be highly relevant to the patient. By assessing outcome measures from multiple perspectives, we will be able to fully capture the functional effects of specialized orthotic care and, at the same time, gain insight in the underlying biomechanical working mechanisms. Additionally, compared to previous research, evidence will be obtained in a large group of adults suffering from leg muscle weakness, caused by many different slowly progressive neuromuscular disorders, which increases the generalizability of results.

Second, participants in the control group will be treated in a diverse sample of usual care centers in which leg orthoses are prescribed throughout the Netherlands. This allows for a broad comparison between specialized orthotic care and orthotic care as applied in current practice, which could be of importance for the improvement of overall quality and efficiency of orthotic care. On the other hand, treatment components of the usual care process are expected to differ amongst centers due to local policies and available facilities. This could complicate the identification of underlying mechanisms of action that could explain differences in treatment outcomes between groups. To enable cautious exploration of potential mechanisms that might lead to beneficial treatment outcomes, which could be useful for future implications, the process of usual orthotic care will be extensively documented.

Finally, a major strength is the economic evaluation that will be performed alongside the study. Since expensive orthotic devices increasingly become available due to technological developments, it is important to not only assess whether orthotic devices are effective, but also whether their additional health effects are worth their additional costs. This study will be the first to provide insights into the cost-effectiveness of orthotic care in NMD in general and of specialized orthotic care compared to usual orthotic care in particular.

In conclusion, this study aims to examine the effectiveness and cost-effectiveness of specialized orthotic care compared to usual orthotic care in adults with NMD. Insights could lead to improvements in the quality of orthotic care resulting in improvements in overall functionality of adults with NMD in daily living. Consequently, insights could lead to a more efficient use of already scarce (healthcare) resources.

For peer review only - http://bmjopen.bmj.com/site/about/guidelines.xhtml

ETHICS AND DISSEMINATION

The study protocol was approved by the Medical Ethics committee of the Amsterdam UMC, location Academic Medical Center (AMC), ABR-number 67268. The study is registered at the Dutch Trial Register (NL7511) and will be performed in accordance with good clinical practice guidelines. An independent monitor of the Amsterdam UMC will monitor the study multiple times during the conduction of the study. Aspects that will be monitored will include inclusion rate, trial master file, informed consent process, inclusion and exclusion criteria, randomization, trial procedures, source data verification, safety reporting and closing and reporting. The investigator will be encouraged to maintain the blinding as far as possible and code breaks should only occur in exceptional circumstances. Important protocol changes will be recorded (a new protocol version number will be assigned) and reported to the Medical Ethics Committee. When patients sign their informed consent, they will receive a participant ID, which will be coupled to all the data collected. Forms will be stored in a locked cabinet to assure anonymity. Only persons involved in the study have access to these forms. Insurance has been taken out for participation of patients in the study. After completion of the study, positive as well as negative or inconclusive results will be submitted to a peer-reviewed journal and presented at national and international scientific conferences. Furthermore, results will be disseminated through other media aimed at a broader audience including patients. The study data sets and codes of the data analysis are available on request. Furthermore, participants will be informed about the results of the study by means of a newsletter.

AUTHOR STATEMENT

M-AB, FSK and FN conceived the study. M-AB, FSK, FN, JMvD and EvD contributed to the study design and methods. M-AB, FSK, FN, JT, RL, VA and EvD participated in logistical planning of the study. EvD wrote the manuscript and is responsible for data acquisition and analysis. All authors conceived, provided feedback and approved the final version of the manuscript.

FUNDING

This study was supported by ZonMw, The Netherlands Organisation for Health Research and Development, grant number: 853001103. The funder will have no role in the execution, analyses, interpretation of the data of the study, or decision to submit results.

COMPETING INTERESTS

None declared.

For peer review only - http://bmjopen.bmj.com/site/about/guidelines.xhtml

REFERENCES

1. Demir YP. Neuromuscular Diseases and Rehabilitation. In: Suzuki T. Neurological Physical Therapy. BoD – Books on Demand 2017.
2. van der Krogt MM, Delp SL, Schwartz MH. How robust is human gait to muscle weakness? *Gait & posture* 2012;36(1):113-9. doi: 10.1016/j.gaitpost.2012.01.017 [published Online First: 2012/03/06]
3. Brehm M, Nollet F, Harlaar J. Energy demands of walking in persons with postpoliomyelitis syndrome: relationship with muscle strength and reproducibility. *Arch Phys Med Rehabil* 2006;87(1):136-40. doi: 10.1016/j.apmr.2005.08.123
4. Menotti F, Felici F, Damiani A, et al. Charcot-Marie-Tooth 1A patients with low level of impairment have a higher energy cost of walking than healthy individuals. *Neuromuscul Disord* 2011;21(1):52-7. doi: 10.1016/j.nmd.2010.09.008 [published Online First: 2010/11/16]
5. Abresch RT, Carter GT, Jensen MP, et al. Assessment of pain and health-related quality of life in slowly progressive neuromuscular disease. *Am J Hosp Palliat Care* 2002;19(1):39-48. doi: 10.1177/104990910201900109 [published Online First: 2002/08/14]
6. Bickerstaffe A, Beelen A, Nollet F. Circumstances and consequences of falls in polio survivors. *J Rehabil Med* 2010;42(10):908-15. doi: 10.2340/16501977-0620 [published Online First: 2010/10/30]
7. Pieterse AJ, Luttikhold TB, de Laat K, et al. Falls in patients with neuromuscular disorders. *Journal of the neurological sciences* 2006;251(1-2):87-90. doi: 10.1016/j.jns.2006.09.008 [published Online First: 2006/11/14]
8. Ahlstrom G, Sjoden PO. Coping with illness-related problems and quality of life in adult individuals with muscular dystrophy. *J Psychosom Res* 1996;41(4):365-76. doi: 10.1016/s0022-3999(96)00191-2 [published Online First: 1996/10/01]
9. Johnson NE, Heatwole CR, Dilek N, et al. Quality-of-life in Charcot-Marie-Tooth disease: the patient's perspective. *Neuromuscul Disord* 2014;24(11):1018-23. doi: 10.1016/j.nmd.2014.06.433 [published Online First: 2014/08/06]
10. Hsu JD, Michael JW, Fisk JR. Atlas of orthoses and assistive devices. In: Surgeons AAoO, ed. 4th edition ed. Philadelphia: American Academy of Orthopedic Surgeons 2008:299-309.
11. ISO. ISO 8549-1 - Vocabulary - Part 1: general terms for external limb prostheses and external orthoses. Genève: International Organization for Standardization, 1989.
12. Hijmans JM, Geertzen JH. Development of clinical guidelines for the prescription of orthoses in patients with neurological disorders in The Netherlands. *Prosthet Orthot Int* 2006;30(1):35-43. doi: 10.1080/03093640500533232 [published Online First: 2006/06/03]
13. Heim S, Jacobs NA, Jensen JS, et al. ISPO consensus conference on poliomyelitis. Consensus Statement and Syndicate Reports. International Society of Prosthetics and Orthotics 1997.
14. van der Wilk D, Dijkstra PU, Postema K, et al. Effects of ankle foot orthoses on body functions and activities in people with floppy paretic ankle muscles: a systematic review. *Clin Biomech (Bristol, Avon)* 2015;30(10):1009-25. doi: 10.1016/j.clinbiomech.2015.09.013 [published Online First: 2015/11/21]
15. McDaid C, Fayter D, Booth A, et al. Systematic review of the evidence on orthotic devices for the management of knee instability related to neuromuscular and central nervous system disorders. *BMJ Open* 2017;7(9):e015927. doi: 10.1136/bmjopen-2017-015927 [published Online First: 2017/09/08]
16. Brehm MA, Beelen A, Doorenbosch CA, et al. Effect of carbon-composite knee-ankle-foot orthoses on walking efficiency and gait in former polio patients. *J Rehabil Med* 2007;39(8):651-7. doi: 10.2340/16501977-0110 [published Online First: 2007/09/27]

For peer review only - http://bmjopen.bmj.com/site/about/guidelines.xhtml

17. Waterval NFJ, Brehm MA, Harlaar J, et al. Description of orthotic properties and effects evaluation of ankle-foot orthoses in non-spastic calf muscle weakness. *J Rehabil Med* 2020 doi: 10.2340/16501977-2642 [published Online First: 2020/01/15]
18. Brehm M, Nollet F. Beenorthesen bij neuromusculaire aandoeningen. Amsterdam: Reed business education 2014.
19. College van Zorgverzekeringen. Procesbeschrijving hulpmiddelenzorg. Den Haag: Nationaal ICT instituut in de Zorg 2009.
20. WHO. International classification of functioning, disability and health: ICF. Genève: World Health Organization, 2001.
21. ISO. ISO 9999 Assistive products for persons with disability – Classification and terminology. Draft International Standard. Genève: International Organization for Standardization, 2010.
22. Hebert JS. Ambulatory KAFOs: A Physiatry Perspective. *J Prosthet Orthot* 2006;18(7 Proceedings):169-74.
23. Brehm M, Bus SA, Harlaar J, et al. A candidate core set of outcome measures based on the International Classification of Functioning, Disability and Health for clinical studies on lower limb orthoses. *Prosthet Orthot Int* 2011;35(3):269-77. doi: 10.1177/0309364611413496 [published Online First: 2011/09/23]
24. Waterval NFJ, Nollet F, Harlaar J, et al. Modifying ankle foot orthosis stiffness in patients with calf muscle weakness: gait responses on group and individual level. *J Neuroeng Rehabil* 2019;16(1):120. doi: 10.1186/s12984-019-0600-2 [published Online First: 2019/10/19]
25. Healy A, Farmer S, Eddison N, et al. A scoping literature review of studies assessing effectiveness and cost-effectiveness of prosthetic and orthotic interventions. *Disabil Rehabil Assist Technol* 2019:1-7. doi: 10.1080/17483107.2018.1523953 [published Online First: 2019/01/18]
26. Vinci P, Gargiulo P. Poor compliance with ankle-foot-orthoses in Charcot-Marie-Tooth disease. *Eur J Phys Rehabil Med* 2008;44(1):27-31. [published Online First: 2008/04/04]
27. Swinnen E, Kerckhofs E. Compliance of patients wearing an orthotic device or orthopedic shoes: A systematic review. *J Bodyw Mov Ther* 2015;19(4):759-70. doi: 10.1016/j.jbmt.2015.06.008 [published Online First: 2015/11/26]
28. Chan AW, Tetzlaff JM, Altman DG, et al. SPIRIT 2013 Statement: Defining standard protocol items for clinical trials. *Ann Intern Med* 2013;158(3):200-207. doi: 10.7326/0003-4819-158-3-201302050-00583 [published Online First: 2013/01/09]
29. Council MR. *Aids to examination of the peripheral nervous system*. Memorandum no 45. London: Her Majesty's Stationary Office 1976.
30. Garby L, Astrup A. The relationship between the respiratory quotient and the energy equivalent of oxygen during simultaneous glucose and lipid oxidation and lipogenesis. *Acta physiologica Scandinavica* 1987;129(3):443-4. doi: 10.1111/j.1365-201x.1987.tb10613.x [published Online First: 1987/03/01]
31. Brehm MA, Verduijn S, Bon J, et al. Comparison of two 6-minute walk tests to assess walking capacity in polio survivors. *J Rehabil Med* 2017;49(9):732-37. doi: 10.2340/16501977-2264 [published Online First: 2017/09/21]
32. Kempen JC, Harlaar J, van der Kooi AJ, et al. Reliability of the walking energy cost test and the six-minute walk test in boys with Duchenne muscular dystrophy. *Neuromuscul Disord* 2014;24(3):216-21. doi: 10.1016/j.nmd.2013.11.015 [published Online First: 2013/12/25]
33. Steenbeek D, Ketelaar M, Galama K, et al. Goal attainment scaling in paediatric rehabilitation: a critical review of the literature. *Dev Med Child Neurol* 2007;49(7):550-6. doi: 10.1111/j.1469-8749.2007.00550.x [published Online First: 2007/06/27]
34. Steenbeek D, Meester-Delver A, Becher JG, et al. The effect of botulinum toxin type A treatment of the lower extremity on the level of functional abilities in children with cerebral palsy: evaluation with goal attainment scaling. *Clin Rehabil* 2005;19(3):274-82. doi: 10.1191/0269215505cr859oa [published Online First: 2005/04/30]
35. Dekkers K, de Viet E, Eilander H, et al. Goal attainment scaling (GAS) in de praktijk [Goal attainment scaling in clinical practice], 2011.

For peer review only - http://bmjopen.bmj.com/site/about/guidelines.xhtml

36. Gandek B, Ware JE, Jr., Aaronson NK, et al. Tests of data quality, scaling assumptions, and reliability of the SF-36 in eleven countries: results from the IQOLA Project. International Quality of Life Assessment. *J Clin Epidemiol* 1998;51(11):1149-58. doi: 10.1016/s0895-4356(98)00106-1 [published Online First: 1998/11/17]
37. Kempen GI, Yardley L, van Haastregt JC, et al. The Short FES-I: a shortened version of the falls efficacy scale-international to assess fear of falling. *Age Ageing* 2008;37(1):45-50. doi: 10.1093/ageing/afm157 [published Online First: 2007/11/23]
38. Krupp LB, LaRocca NG, Muir-Nash J, et al. The fatigue severity scale. Application to patients with multiple sclerosis and systemic lupus erythematosus. *Arch Neurol* 1989;46(10):1121-3. doi: 10.1001/archneur.1989.00520460115022 [published Online First: 1989/10/01]
39. Wessels RD, De Witte LP. Reliability and validity of the Dutch version of QUEST 2.0 with users of various types of assistive devices. *Disabil Rehabil* 2003;25(6):267-72. doi: 10.1080/0963828021000031197 [published Online First: 2003/03/08]
40. Kanters TA, Bouwmans CAM, van der Linden N, et al. Update of the Dutch manual for costing studies in health care. *PloS one* 2017;12(11):e0187477. doi: 10.1371/journal.pone.0187477 [published Online First: 2017/11/10]
41. Versteegh MM, Vermeulen KM, Evers SM, et al. Dutch Tariff for the Five-Level Version of EQ-5D. *Value health* 2016;19(4):343-52. doi: 10.1016/j.jval.2016.01.003 [published Online First: 2016/06/22]

For peer review only

For peer review only - http://bmjopen.bmj.com/site/about/guidelines.xhtml

Figure legends

Figure 1*: Overview of the study design.*

For peer review only

For peer review only - http://bmjopen.bmj.com/site/about/guidelines.xhtml

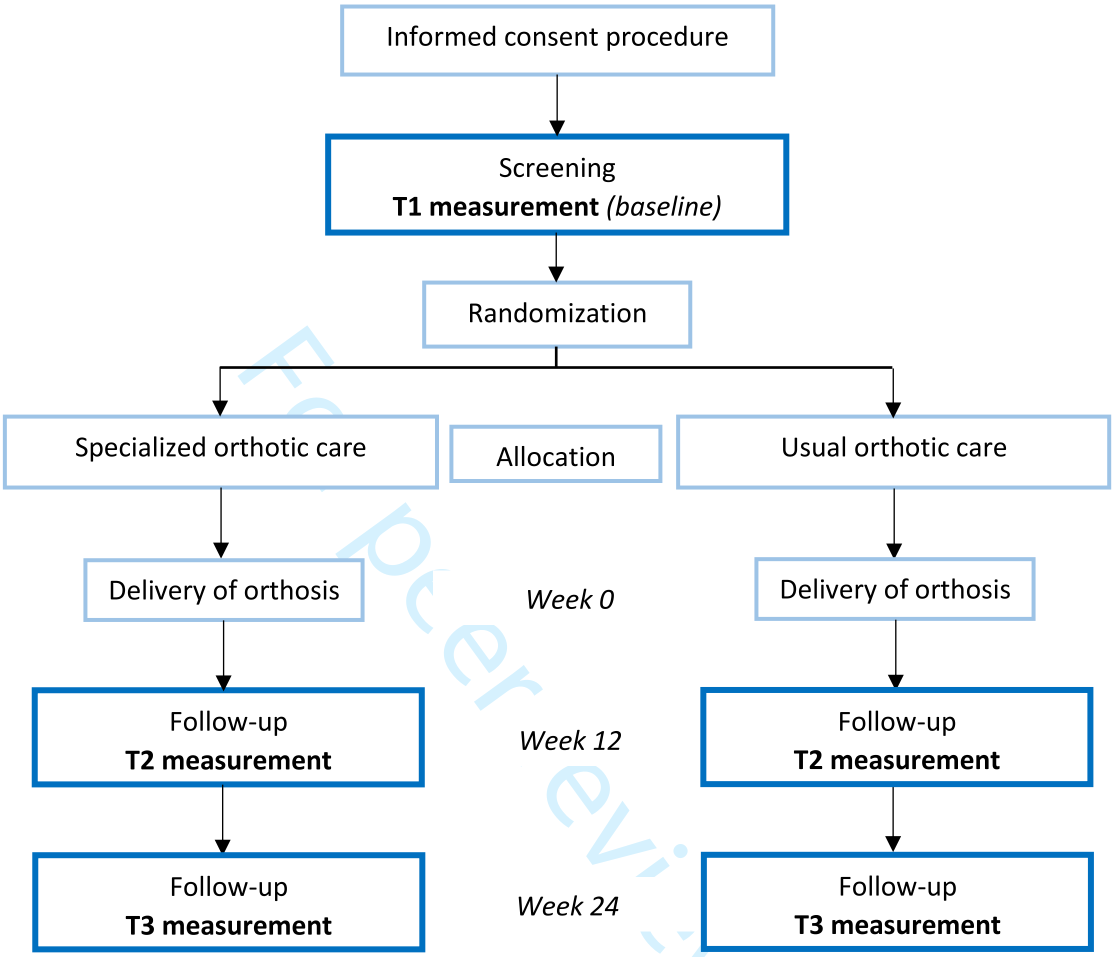
Informed consent procedure
Screening
T1 measurement *(baseline)*
Randomization
Specialized orthotic care
Allocation
Usual orthotic care
Delivery of orthosis
Week 0
Delivery of orthosis
Follow-up
T2 measurement
Week 12
Follow-up
T2 measurement
Follow-up
T3 measurement
Week 24
Follow-up
T3 measurement

Reporting checklist for protocol of a clinical trial.

Based on the SPIRIT guidelines.

		Reporting Item	Page Number
**Administrative information**			
Title	#1	Descriptive title identifying the study design, population, interventions, and, if applicable, trial acronym	1
Trial registration	#2a	Trial identifier and registry name. If not yet registered, name of intended registry	2
Trial registration: data set	#2b	All items from the World Health Organization Trial Registration Data Set	1-12
Protocol version	#3	Date and version identifier	1
Funding	#4	Sources and types of financial, material, and other support	12
Roles and responsibilities: contributorship	#5a	Names, affiliations, and roles of protocol contributors	1, 12
Roles and responsibilities: sponsor contact information	#5b	Name and contact information for the trial sponsor	1
Roles and responsibilities: sponsor and funder	#5c	Role of study sponsor and funders, if any, in study design; collection, management, analysis, and interpretation of data; writing of the report; and the decision to submit the report for publication, including whether they will have ultimate authority over any of these activities	12
Roles and responsibilities: committees	#5d	Composition, roles, and responsibilities of the coordinating centre, steering committee, endpoint adjudication committee, data management team, and other individuals or groups overseeing the trial, if applicable (see Item 21a for data monitoring committee)	12
**Introduction**			
Background and rationale	#6a	Description of research question and justification for undertaking the trial, including summary of relevant studies	4

For peer review only

		(published and unpublished) examining benefits and harms for each intervention	
Background and rationale: choice of comparators	#6b	Explanation for choice of comparators	5
Objectives	#7	Specific objectives or hypotheses	5
Trial design	#8	Description of trial design including type of trial (eg, parallel group, crossover, factorial, single group), allocation ratio, and framework (eg, superiority, equivalence, non-inferiority, exploratory)	6
**Methods: Participants, interventions, and outcomes**			
Study setting	#9	Description of study settings (eg, community clinic, academic hospital) and list of countries where data will be collected. Reference to where list of study sites can be obtained	6
Eligibility criteria	#10	Inclusion and exclusion criteria for participants. If applicable, eligibility criteria for study centres and individuals who will perform the interventions (eg, surgeons, psychotherapists)	6
Interventions: description	#11a	Interventions for each group with sufficient detail to allow replication, including how and when they will be administered	7
Interventions: modifications	#11b	Criteria for discontinuing or modifying allocated interventions for a given trial participant (eg, drug dose change in response to harms, participant request, or improving / worsening disease)	n/a
Interventions: adherance	#11c	Strategies to improve adherence to intervention protocols, and any procedures for monitoring adherence (eg, drug tablet return; laboratory tests)	n/a
Interventions: concomitant care	#11d	Relevant concomitant care and interventions that are permitted or prohibited during the trial	n/a
Outcomes	#12	Primary, secondary, and other outcomes, including the specific measurement variable (eg, systolic blood pressure), analysis metric (eg, change from baseline, final value, time to event), method of aggregation (eg, median, proportion),	8

For peer review only

		and time point for each outcome. Explanation of the clinical relevance of chosen efficacy and harm outcomes is strongly recommended	
Participant timeline	#13	Time schedule of enrolment, interventions (including any run-ins and washouts), assessments, and visits for participants. A schematic diagram is highly recommended (see Figure)	6
Sample size	#14	Estimated number of participants needed to achieve study objectives and how it was determined, including clinical and statistical assumptions supporting any sample size calculations	6
Recruitment	#15	Strategies for achieving adequate participant enrolment to reach target sample size	6
**Methods: Assignment of interventions (for controlled trials)**			
Allocation: sequence generation	#16a	Method of generating the allocation sequence (eg, computer-generated random numbers), and list of any factors for stratification. To reduce predictability of a random sequence, details of any planned restriction (eg, blocking) should be provided in a separate document that is unavailable to those who enrol participants or assign interventions	6-7
Allocation concealment mechanism	#16b	Mechanism of implementing the allocation sequence (eg, central telephone; sequentially numbered, opaque, sealed envelopes), describing any steps to conceal the sequence until interventions are assigned	6-7
Allocation: implementation	#16c	Who will generate the allocation sequence, who will enrol participants, and who will assign participants to interventions	6
Blinding (masking)	#17a	Who will be blinded after assignment to interventions (eg, trial participants, care providers, outcome assessors, data analysts), and how	7
Blinding (masking): emergency unblinding	#17b	If blinded, circumstances under which unblinding is permissible, and procedure for revealing a participant's allocated intervention during the trial	12

For peer review only

**Methods: Data collection, management, and analysis**			
Data collection plan	#18a	Plans for assessment and collection of outcome, baseline, and other trial data, including any related processes to promote data quality (eg, duplicate measurements, training of assessors) and a description of study instruments (eg, questionnaires, laboratory tests) along with their reliability and validity, if known. Reference to where data collection forms can be found, if not in the protocol	8-10
Data collection plan: retention	#18b	Plans to promote participant retention and complete follow-up, including list of any outcome data to be collected for participants who discontinue or deviate from intervention protocols	12
Data management	#19	Plans for data entry, coding, security, and storage, including any related processes to promote data quality (eg, double data entry; range checks for data values). Reference to where details of data management procedures can be found, if not in the protocol	12
Statistics: outcomes	#20a	Statistical methods for analysing primary and secondary outcomes. Reference to where other details of the statistical analysis plan can be found, if not in the protocol	10
Statistics: additional analyses	#20b	Methods for any additional analyses (eg, subgroup and adjusted analyses)	10
Statistics: analysis population and missing data	#20c	Definition of analysis population relating to protocol non-adherence (eg, as randomised analysis), and any statistical methods to handle missing data (eg, multiple imputation)	10
**Methods: Monitoring**			
Data monitoring: formal committee	#21a	Composition of data monitoring committee (DMC); summary of its role and reporting structure; statement of whether it is independent from the sponsor and competing interests; and reference to where further details about its charter can be found, if not in the protocol. Alternatively, an explanation of why a DMC is not needed	12

For peer review only

Data monitoring: interim analysis	#21b	Description of any interim analyses and stopping guidelines, including who will have access to these interim results and make the final decision to terminate the trial	12
Harms	#22	Plans for collecting, assessing, reporting, and managing solicited and spontaneously reported adverse events and other unintended effects of trial interventions or trial conduct	6
Auditing	#23	Frequency and procedures for auditing trial conduct, if any, and whether the process will be independent from investigators and the sponsor	12
**Ethics and dissemination**			
Research ethics approval	#24	Plans for seeking research ethics committee / institutional review board (REC / IRB) approval	12
Protocol amendments	#25	Plans for communicating important protocol modifications (eg, changes to eligibility criteria, outcomes, analyses) to relevant parties (eg, investigators, REC / IRBs, trial participants, trial registries, journals, regulators)	12
Consent or assent	#26a	Who will obtain informed consent or assent from potential trial participants or authorised surrogates, and how (see Item 32)	6
Consent or assent: ancillary studies	#26b	Additional consent provisions for collection and use of participant data and biological specimens in ancillary studies, if applicable	n/a
Confidentiality	#27	How personal information about potential and enrolled participants will be collected, shared, and maintained in order to protect confidentiality before, during, and after the trial	12
Declaration of interests	#28	Financial and other competing interests for principal investigators for the overall trial and each study site	12
Data access	#29	Statement of who will have access to the final trial dataset, and disclosure of contractual agreements that limit such access for investigators	12
Ancillary and post trial care	#30	Provisions, if any, for ancillary and post-trial care, and for compensation to those who suffer harm from trial participation	12

For peer review only

Dissemination policy: trial results	#31a	Plans for investigators and sponsor to communicate trial results to participants, healthcare professionals, the public, and other relevant groups (eg, via publication, reporting in results databases, or other data sharing arrangements), including any publication restrictions	12
Dissemination policy: authorship	#31b	Authorship eligibility guidelines and any intended use of professional writers	12
Dissemination policy: reproducible research	#31c	Plans, if any, for granting public access to the full protocol, participant-level dataset, and statistical code	12
**Appendices**			
Informed consent materials	#32	Model consent form and other related documentation given to participants and authorised surrogates	supplement 2
Biological specimens	#33	Plans for collection, laboratory evaluation, and storage of biological specimens for genetic or molecular analysis in the current trial and for future use in ancillary studies, if applicable	n/a

The SPIRIT checklist is distributed under the terms of the Creative Commons Attribution License CC-BY-ND 3.0. This checklist was completed on 22. April 2020 using https://www.goodreports.org/, a tool made by the EQUATOR Network in collaboration with Penelope.ai

For peer review only

Subject information

Appendix: consent form subject

‘Specialized orthotic care for people with leg muscle weakness’

- I have read the information letter. I was also able to ask questions. My questions have been answered sufficiently. I have had enough time to decide whether or not to participate.

- I understand that participation is voluntary. I also know that I may decide at any time to not participate or to stop participating in the study. Without having to provide any reason.

- I give consent for my general practitioner to be informed of my participation in this study.

- I give consent to information being requested from my specialist(s) treating me about my medical history.

- I give consent to collect and use my data for answering the research question in this study.

- I know that for study monitoring purposes some individuals could have access to all my data. Those people are listed in this information letter. I consent to that access by these persons.

- I give consent for my general practitioner and/or treating specialist to be informed of unexpected findings which are (may be) of interest for my health.

- I **□ give**
  □ do not give
  consent for the further storage of my personal data for 15 years and retention for future research into the area of my disorder and the method of treatment.

- I **□ give**
  □ do not give
  consent to being contacted again after this study for a follow-up study.

For peer review only - http://bmjopen.bmj.com/site/about/guidelines.xhtml

Subject information

- I want to participate in this study.

Name of subject: …………………………………………………………………………..
Signature: Date : __ / __ / __

I certify that I have fully informed this subject about the said study.

If information becomes known during the study that could influence the consent of the subject, I will inform him/her of this on time.

Name of investigator :……………………………………………………………………
Signature: Date: __ / __ / __

The subject will receive a complete information letter, together with a signed version of the informed consent form.

For peer review only - http://bmjopen.bmj.com/site/about/guidelines.xhtml

BMJ Open

Specialized orthotic care to improve functioning in adults with neuromuscular disorders: protocol of a prospective randomized open label blinded end-point study.

Journal:	*BMJ Open*
Manuscript ID	bmjopen-2020-039683.R2
Article Type:	Protocol
Date Submitted by the Author:	19-Oct-2020
Complete List of Authors:	van Duijnhoven, Elza; Amsterdam UMC, University of Amsterdam, Department of Rehabilitation Medicine, Amsterdam Movement Sciences<br>Koopman, Fieke; Amsterdam UMC, University of Amsterdam, Department of Rehabilitation Medicine, Amsterdam Movement Sciences<br>Tuijtelaars, Jana; Amsterdam UMC, University of Amsterdam, Department of Rehabilitation Medicine, Amsterdam Movement Sciences<br>Altmann, Viola; Klimmendaal, Rehabilitation center<br>Lagrand, Rimke; Amsterdam UMC, University of Amsterdam, Department of Rehabilitation Medicine, Amsterdam Movement Sciences<br>van Dongen, Johanna; Vrije Universiteit Amsterdam, Department of Health Sciences, Faculty of Science<br>Nollet, Frans; Amsterdam UMC, University of Amsterdam, Department of Rehabilitation Medicine, Amsterdam Movement Sciences<br>Brehm, Merel-Anne; Amsterdam UMC, University of Amsterdam, Department of Rehabilitation Medicine, Amsterdam Movement Sciences
**Primary Subject Heading**:	Rehabilitation medicine
Secondary Subject Heading:	Neurology
Keywords:	Neuromuscular disease < NEUROLOGY, REHABILITATION MEDICINE, Protocols & guidelines < HEALTH SERVICES ADMINISTRATION & MANAGEMENT

SCHOLARONE™
Manuscripts

I, the Submitting Author has the right to grant and does grant on behalf of all authors of the Work (as defined in the below author licence), an exclusive licence and/or a non-exclusive licence for contributions from authors who are: i) UK Crown employees; ii) where BMJ has agreed a CC-BY licence shall apply, and/or iii) in accordance with the terms applicable for US Federal Government officers or employees acting as part of their official duties; on a worldwide, perpetual, irrevocable, royalty-free basis to BMJ Publishing Group Ltd ("BMJ") its licensees and where the relevant Journal is co-owned by BMJ to the co-owners of the Journal, to publish the Work in this journal and any other BMJ products and to exploit all rights, as set out in our licence.

The Submitting Author accepts and understands that any supply made under these terms is made by BMJ to the Submitting Author unless you are acting as an employee on behalf of your employer or a postgraduate student of an affiliated institution which is paying any applicable article publishing charge ("APC") for Open Access articles. Where the Submitting Author wishes to make the Work available on an Open Access basis (and intends to pay the relevant APC), the terms of reuse of such Open Access shall be governed by a Creative Commons licence – details of these licences and which Creative Commons licence will apply to this Work are set out in our licence referred to above.

Other than as permitted in any relevant BMJ Author's Self Archiving Policies, I confirm this Work has not been accepted for publication elsewhere, is not being considered for publication elsewhere and does not duplicate material already published. I confirm all authors consent to publication of this Work and authorise the granting of this licence.

For peer review only

Specialized orthotic care to improve functioning in adults with neuromuscular disorders: protocol of a prospective randomized open label blinded end-point study.

Elza van Duijnhoven[1], Fieke Sophia Koopman[1], Jana Tuijtelaars[1], Viola Altmann[2], Rimke Lagrand[1], Johanna Maria van Dongen[3], Frans Nollet[1], Merel-Anne Brehm[1]

[1]Amsterdam UMC, University of Amsterdam, Department of Rehabilitation Medicine, Amsterdam Movement Sciences, Meibergdreef 9, Amsterdam, the Netherlands

[2]Klimmendaal, Rehabilitation center, Arnhem, the Netherlands.

[3]Department of Health Sciences, Faculty of Science, Vrije Universiteit Amsterdam ,Amsterdam Movement Sciences, Amsterdam, the Netherlands

*Correspondence to: Elza van Duijnhoven
Department of Rehabilitation Medicine
Amsterdam UMC, location Academic Medical Center
Meibergdreef 9
1105 AZ, Amsterdam
The Netherlands
Email: e.vanduijnhoven@amsterdamumc.nl
Telephone: +31 20 566 6915
Fax: +31 20 5669154

Issue date: 16-10-2020
Word count: 4000

For peer review only

For peer review only - http://bmjopen.bmj.com/site/about/guidelines.xhtml

ABSTRACT

Introduction

People suffering from leg muscle weakness caused by neuromuscular disorders (NMD) are often provided with leg orthoses to reduce walking problems such as increased walking effort, diminished walking speed, reduced balance and falls. However, evidence for the effectiveness of leg orthoses to improve walking in this patient group is limited and there is an absence of standardized practice in orthotic prescription. In 2012 a Dutch multidisciplinary guideline was developed aimed to standardize the orthotic treatment process in NMD. Although application of the guideline in expert centers (specialized orthotic care) seems beneficial regarding clinical effectiveness, larger studies are necessary to confirm results and investigate cost-effectiveness. Therefore, this study aims to examine the effectiveness and cost-effectiveness of specialized orthotic care compared to usual orthotic care in adults with slowly progressive NMD.

Methods and analysis

A prospective randomized open label blinded end-point study will be performed, in which 70 adults with slowly progressive NMD are randomly assigned to specialized orthotic care (intervention) or usual orthotic care (control). Outcome measures are assessed at baseline and at 3 and 6 months follow-up. The primary endpoints are gross walking energy cost (J/kg/m) assessed during a 6-minute walk test and achievement of personal goals, measured with the goal attainment scale. Secondary endpoints include walking speed, gait biomechanics, stability, physical functioning, falls and fear of falling, perceived fatigue, and satisfaction. For the economic evaluation, societal costs and health-related quality of life will be assessed using cost questionnaires and the EQ-5D-5L, retrospectively.

Ethics and dissemination

The study is registered in the Dutch trial register (NL 7511) and the protocol has been approved by the Medical Ethics Committee of the Academic Medical Center in Amsterdam. Results will be presented at national and international scientific conferences and disseminated through peer-reviewed journals and media aimed at a broad audience including patients.

Keywords

Neuromuscular diseases; orthotic devices; specialized orthotic care; cost-effectiveness, walking energy cost, goal attainment scale; RCT.

For peer review only - http://bmjopen.bmj.com/site/about/guidelines.xhtml

ARTICLE SUMMARY

Strengths and limitations of this study

- A broad range of outcome measures, both objective and self-reported, will be collected, resulting in a unique dataset of both functional effects, underlying biomechanical working mechanisms and costs.
- The two primary outcome measures to evaluate functional effects include walking energy cost and achievement of personal goals measured with the Goal Attainment Scale, which is chosen as an additional primary outcome measure to capture the diversity of orthotic goals relevant to the individual.
- Evidence will be obtained in a large group of adults with different slowly progressive neuromuscular disorders, which increases the generalizability of results.
- An economic evaluation will be performed alongside the study to provide insights into the cost-effectiveness of specialized orthotic care versus usual orthotic care.
- To account forthe expected variability in treatment components of usual orthotic care, which is a possible limitation that could complicate the identification of underlying mechanisms of action between treatments, the process of usual orthotic care will be extensively documented.

For peer review only

For peer review only - http://bmjopen.bmj.com/site/about/guidelines.xhtml

INTRODUCTION

People with slowly progressive neuromuscular disorders (NMD), such as post-polio syndrome and Charcot Marie Tooth disease, frequently suffer from leg muscle weakness[1]. Leg muscle weakness will change the gait pattern[2], causing walking problems such as increased walking effort[3 4], diminished walking speed[3 4], pain[5], balance problems and falls[6 7]. These walking problems may restrict the patients' performance of daily physical activities[8] and negatively affect their independency and quality of life[8 9]. Due to the progressive nature of NMD, walking problems will gradually increase over time[9], which will further negatively affect physical mobility, independency and quality of life.

Leg orthoses are provided to reduce walking problems in people with NMD[10]. A leg orthosis is an "externally applied medical device encompassing (part of) the leg and foot, used to modify the structural and functional characteristics of the neuromuscular and skeletal systems"[11]. In general, an ankle-foot orthosis (AFO) is provided in case of distal leg muscle weakness, which often includes weakness of the foot dorsiflexors and plantarflexors[10 12]. Accordingly, the AFO should support foot clearance during swing by restricting plantarflexion and compensate for plantarflexor weakness by restricting dorsiflexion during late stance[10]. Knee-ankle-foot orthoses (KAFOs) are provided for (additional) proximal leg muscle weakness, particularly weakness of the quadriceps, to stabilize the knee joint during the stance phase of gait and allow safe weight-bearing[10 13].

In previous research, various types of AFOs for calf muscle weakness[14] and KAFOs for quadriceps weakness have been found to improve walking in people with NMD[15]. However, the strength of the evidence is rather limited due to a lack of proper study designs, relatively small sample sizes, and heterogeneity in types of leg orthoses and control conditions studied. Accordingly, there is a lack of evidence-based and standardized guidance on the application of leg orthoses. Furthermore, many different types of leg orthoses are being prescribed in current orthotic practice in NMD, varying largely in orthotic properties and effectiveness, with both good and suboptimal treatment outcomes in terms of improving walking[16 17].

In 2012, a Dutch multidisciplinary guideline for leg orthoses in adults with slowly progressive NMD was developed with the aim to standardize the treatment process and improve treatment outcomes[18]. The guideline was developed according to proposed national and international frameworks[19 20 21] and compromises the entire process of orthotic treatment in systematically divided steps, as well as treatment algorithms for the selection of leg orthoses. According to the guideline, the care need should be individually characterized in terms of the personal health problems, goals and gait deviations to be addressed, caused by the underlying impairments. Based on the care need, the orthotic goals should be defined and matched with the orthosis design. Gait training after delivery of the orthosis after is essential to maximize effectiveness[22]. Finally, it is important to systematically evaluate the effectiveness as well as user experiences to ensure the functionality of the orthosis[23].

At this moment, the guideline is not widely applied throughout the Netherlands, as it requires sufficient expertise of the multidisciplinary care team involved and facilities for advanced 3D-gait analysis and gait training. However, prescribing leg orthoses according to the guideline (i.e. specialized orthotic care) seems promising in terms of clinical effectiveness as recently shown in two uncontrolled trials[16 24]. In individuals with calf muscle weakness due to NMD, individually stiffness-optimized AFOs have been shown to reduce walking effort to a much greater extent compared to AFOs prescribed in usual orthotic care[24]. Furthermore, in polio survivors[16], the increment in walking effort with KAFOs

For peer review only - http://bmjopen.bmj.com/site/about/guidelines.xhtml

prescribed in specialized orthotic care was reduced with 18% towards normative values, when compared to usual care KAFOs. At this moment, the effectiveness of leg orthoses prescribed within specialized orthotic care on functioning needs to be investigated in a larger group of individuals with slowly progressive NMD in comparison to usual orthotic care to strengthen evidence for the possible benefit.

While good-quality studies are needed to strengthen the evidence for the effectiveness of specialized orthotic care in NMD, it is also imperative to investigate whether the treatment is cost-effective, which is currently unknown. Since new and expensive orthotic devices increasingly become available, it is important to assess their associated costs[25]. Also, at this moment, an important problem in usual orthotic care concerns the low compliance of patients wearing their devices[26], which in turn leads to an inefficient use of healthcare resources[27]. Optimally matching orthoses with the personal needs and goals of the patient, according to the guideline, could lead to gains in clinical effectiveness and subsequently a higher compliance. In this respect, specialized orthotic care could improve the efficiency of (healthcare) resource, but this has not yet been investigated.

The aim of this study is to examine the effectiveness and cost-effectiveness of specialized orthotic care compared to usual orthotic care on functioning in adults with NMD. We hypothesize that specialized orthotic care is more effective in terms of reducing walking effort and achieving personal goals when compared to usual orthotic care. Furthermore, specialized orthotic care is expected to be cost-effective compared with usual orthotic care from a societal and healthcare perspective.

For peer review only

For peer review only - http://bmjopen.bmj.com/site/about/guidelines.xhtml

METHODS AND ANALYSIS

Study design

The study is designed as a randomized open label blinded end-point study that is prospectively registered at the Dutch Trial Register under number NL7511. The trial protocol was developed according to the SPIRIT guidelines[28] (supplementary file 1). Recruitment started in March 2019 and is foreseen to end at December 2021. Measurements are conducted at baseline (T1) and 3 and 6 months after orthotic treatment is given (T2 and T3, respectively). To determine the cost-effectiveness, an economic evaluation is performed alongside the study. An overview of the study design is shown in Figure 1.

Study population

We intend to include 70 adults with slowly progressive NMD (such as Charcot-Marie-Tooth disease, post-polio syndrome, inclusion body myositis or myotonic dystrophy) or peripheral nerve injury who have non-spastic leg muscle weakness. For eligibility, patients may or may not use an orthosis provided in usual care at the time of recruitment. Patients will be recruited from rehabilitation centers and hospitals throughout the Netherlands and through the Dutch Association for Neuromuscular Diseases. If a patient is willing to participate, a screening visit is planned. During the screening visit, written informed consent (supplementary file 2) is obtained and the investigator, in close collaboration with a rehabilitation physician, will check the in- and exclusion criteria.

Inclusion criteria are: (1) minimum age of 18 years, (2) weakness of the calf muscles (i.e. Medical Research Council (MRC) scale[29] score < 5 or not being able to make a heel-rise on one leg > 3 times) and/or weakness of the quadriceps (i.e. MRC score < 5) (3) experiencing walking problems such as increased walking effort, pain and/or impaired balance during standing and/or walking, (4) able to walk for 6 minutes at comfortable speed with or without assistive device (e.g. cane, crutch, walker), (5) indicated for an orthosis based upon physical examination and 3D gait analysis, and (6) motivated to use an orthosis. An exclusion criterion is: (1) insufficient mastery of the Dutch language.

Sample size

The sample size of this study is based on a power analysis of the expected differences in walking effort (defined as gross walking energy cost) and the achievement of personal treatment goals between the intervention and control group. According to previous studies[16 17], a difference in change in walking energy cost of 0.60 J/kg/m from baseline to 6 months post-treatment is expected between usual orthotic care and specialized orthotic care, where a difference of at least 0.45 J/kg/m is considered as clinical relevant[3]. Based on an intention-to-treat analysis, alpha of 0.05, power of 80%, and using an estimated correlation coefficient of the repeated measures of 0.76 and standard deviation of 0.90 J/kg/m, 30 patients per treatment group are necessary. The goal is to include 70 patients allowing for a 15% drop out. This sample size also allows the detection of 1 point difference in personal achievement goal scores (Based on an intention-to-treat analysis, alpha of 0.05, power of 80%, and using an estimated correlation coefficient of the repeated measures of 0.70).

Randomization and blinding

After baseline measurement (T1), participants will be randomly assigned in a 1:1 ratio to receive specialized orthotic care (intervention group) or usual orthotic care (control group). The randomization

For peer review only - http://bmjopen.bmj.com/site/about/guidelines.xhtml

scheme will be computer-generated in Castor (Castor EDC, Amsterdam, the Netherlands) and uses blocks of random sequences with variable sizes (2, 4 and 6). Patients will be stratified by disease severity defined as distal leg weakness versus (distal and) proximal leg weakness, respectively. Outcomes will be assessed by a blinded and independent assessor. When patients are informed of group allocation, they will be instructed not to reveal this to the outcome assessor.

Intervention

Specialized orthotic care

Patients in the intervention group will receive specialized orthotic care at an expert center that has implemented the Dutch guideline for leg orthoses[18] and has facilities for 3D gait analysis, gait training and fabricating custom-made leg orthoses. For the orthotic care process, protocols of the guideline will be used and treatment integrity checks will be performed to ensure compliance with the guideline.

First, the health problems and goals of the patient will assessed by means of an interview, questionnaires, physical examination and 3D gait analysis. Based on these assessments, the multidisciplinary care team will identify how the patients' problems and goals are best matched to an orthotic solution. The desired level of functioning for the patient and the required orthotic functions will be described in a care plan. Accordingly, the orthosis will be custom fabricated by an orthotic technician. After delivery of the orthosis, instructions on use and maintenance will be given to the patient and, when indicated, the patient receives gait training supervised by an experienced physiotherapist. Finally, after 3 months of using the orthosis, experiences of the patient as well as the desired level of functioning and orthotic functions will be evaluated. When needed, the orthosis will be adjusted based on the evaluation. The provision process will be extensively documented for each participant individually by an investigator who is not blinded.

Usual orthotic care

Participants in the control group will receive usual orthotic care from a rehabilitation physician at their regular care center. Treatment can vary across centers and may include provision of orthopedic shoes, provision of off-the-shelf or custom-made AFOs or provision of off-the-shelf or custom-made KAFOs according to the discretion of the practitioners and local policy of the center. For participants who already use an orthosis at baseline, usual orthotic care will concern the provision of a new orthosis which may be identical of different from the orthosis being used. The provision process in the usual care centers will be extensively documented for each participant individually by an investigator who is not blinded.

Outcome measures

Relevant demographic variables, anthropometrics and clinical characteristics will be measured at baseline (T1). Clinical characteristics to be evaluated include: 1) manually assessed muscle strength of the left and right hip flexors and extensors, hip abductors and adductors, knee flexors and extensors, ankle plantar and dorsal flexors and ankle invertors and evertors, scored according to the MRC scale, and 2) passive range of motion of the hip, knee and ankle joints (left and right). Furthermore, 3) occurrence of joint deformities and 4) impairments in sensory function will be evaluated.

Primary outcomes and secondary outcomes (described below) will be assessed at baseline (T1) and at 3 and 6 months after orthotic treatment (T2 and T3, respectively). Additionally, orthotic properties

For peer review only - http://bmjopen.bmj.com/site/about/guidelines.xhtml

(such as type, weight, material and orthotic functions) and adverse events due to the use of the device (such as pressure sores, pain, muscle soreness) will be documented. The use of any medication during the study will be monitored at all measurement time points and documented in the cost questionnaires. All collected outcomes will be entered into a Castor database. An overview of all outcomes per measurement visit is given in Table 1.

Table 1*: Overview of outcomes per measurement session.*

		Baseline	Follow-up	
		**T1 measurement** *screening*	**T2 measurement** *(12 weeks after delivery)*	**T3 measurement** *(24 weeks after delivery)*
**Primary outcomes**				
Walking energy cost	*6MWT*	X	X	X
Personal goals	*GAS*	Setting goals	X	X
**Secondary outcomes**				
Walking speed	*6MWT*	X	X	X
Gait biomechanics	*3DGA*	X*	X	X
Stability	*NRS*	X	X	X
Physical functioning	*SF36-PF*	X	X	X
Fear of falling	*FES*	X	X	X
Fall rate	*Questionnaire*	X	X	X
Fatigue	*FSS*	X	X	X
Satisfaction	*D-Quest*	X**	X	X
**Additional outcomes**				
Demographics	*Intake*	X		
Anthropometrics	*Physical exam*	X		
Muscle strength	*Physical exam*	X		
Joint passive range of motion	*Physical exam*	X		
Sensory function	*Physical exam*	X		
Orthotic properties	*CRF*	X**	X	X
Adverse events	*CRF*		X	X
**Economic evaluation**				
Resource use***	*Cost questionnaire*	X	X	X
Health-related quality of life***	*EQ-5D-5L*	X	X	X

*Gait analysis conditions at baseline that will be used for statistical analysis concern walking with shoes only or walking with the old orthosis (in case a participant uses an orthosis at baseline).
**Outcomes will only be assessed in case a participant uses an orthosis at baseline.
***Outcomes for the economic evaluation will also be assessed directly after delivery of the orthosis.

6MWT = 6-minute walk test, GAS = Goal Attainment Scale, 3DGA = 3-dimensional gait analysis, NRS = numeric rating scale, SF36-PF = physical functioning scale of the SF36, FES = Falls efficacy scale, FSS = Fatigue severity scale, D-Quest = Dutch version of the Quebec User Evaluation of Satisfaction with Assistive Technology, CRF = clinical report form, EQ-5D-5L = 5-level version of EuroQol 5D.

Primary outcomes

Primary outcomes are walking energy cost and the achievement of personal treatment goals. Walking energy cost will be determined during a 6-minute walk test (6MWT) in which participants walk at a self-selected comfortable speed on an indoor oval track in their preferred walking direction (which will be kept similar over measurement time points). Simultaneously, oxygen uptake (VO_2) and the

For peer review only - http://bmjopen.bmj.com/site/about/guidelines.xhtml

respiratory exchange ratio (RER) will be measured breath-by-breath with the Cosmed K5 portable gas analysis system (Cosmed, Rome, Italy). Mean VO_2, RER and walking speed values will be obtained at steady state from the last 3 minutes of the 6MWT using a custom-written Matlab script (V.2019; MathWorks, Natick, Massachusetts, USA). From these outcomes, walking energy cost in J/kg/m will be calculated by using the following formula: ((4.940 x RER) + 16.040) x VO_2)/ walking speed where VO_2 is in ml/kg/min[30]. The assessment of walking energy cost has previously been shown to be reliable in patients with NMD[31 32].

The achievement of personal treatment goals will be quantified with the Goal Attainment Scale (GAS) to capture the diversity of orthotic treatment goals important to the individual patient[33]. At baseline, two goals that are highly relevant to the individual will be determined with the patient based on what they wish to achieve in daily life in terms of activities and participation according to the International Classification of Functioning, Disability and Health (ICF) framework[20]. Subsequently, personal GAS scales will be determined by creating six distinct levels of outcome ranging from -3 to +2, where the desired attainment goal of the patient is defined as 0 and the current situation is defined as -2. Achievement of the goals will be scored at T2 and T3 as -3 = worsened, -2 = unchanged, -1 = somewhat less than expected, 0 = expected outcome, +1 = somewhat more than expected and +2 = much more than expected. Improvements of at least 2 points will be regarded as clinical relevant[34]. The investigator defining and scoring the GAS, followed a course in applying GAS within a rehabilitation context[35].

Secondary outcomes

Secondary outcomes include walking speed (measured during the 6MWT), gait biomechanics (explained below), perceived stability during walking (assessed with a 11-point numeric rating scale (NRS)), perceived physical functioning (assessed with the Short-Form Health Survey physical functioning scale (SF36-PF)[36]), frequency of falls and fear of falling (assessed with the short version of the Falls Efficacy Scale (FES)[37], perceived fatigue (assessed with the Fatigue Severity Scale (FSS)[38]) and satisfaction with the orthosis (assessed with the Dutch version of the Quebec User Evaluation of Satisfaction with Assistive Technology (D-QUEST)[39] added with self-designed items).

Gait biomechanics

Gait biomechanics will be assessed with a 100Hz 12-camera 3D motion capture system (VICON MX 1.3) and two adjacent 1000Hz force plates (OR6-7; AMTI, Watertown, Marssachusetts, USA). Preparations include placement of reflective markers on the body according to the Plug-In Gait model. After a static calibration, participants will be asked to walk along a 12-m long walkway. Conditions that will be assessed include barefoot walking (T1), walking with the current orthosis if applicable (T1) and walking with the new orthosis (T2 and T3). Per condition, at least three valid trials will be used for analysis containing a clear stance phase on the force plate for both feet and full visibility of all markers during the gait cycle. Joint angles, net joint moments and joint powers around the hip, knee and ankle will be calculated per trial and averaged over three trials. Spatiotemporal gait parameters (such as step length and step width) and relevant kinematic and kinetic variables (such as maximal ankle dorsiflexion angle, peak ankle power, knee angle and knee moment at midstance, hip flexion angle and maximal hip, knee and ankle angle during swing and progression of the center of pressure) will be obtained from these averaged data and used for analysis.

For peer review only - http://bmjopen.bmj.com/site/about/guidelines.xhtml

Economic evaluation

The economic evaluation will be performed from a societal and a healthcare perspective. When the societal perspective is applied intervention costs (costs directly related to the delivery of the orthosis) healthcare costs (costs related to visits to general practitioners, medical specialists, and/or therapists, medication use and assistive devices) informal care costs, unpaid productivity costs, as well as costs related to productivity losses due to being absent from work (absenteeism) and productivity losses due to reduced productivity while being at work (presenteeism) will be included and assessed with a cost questionnaire. When the healthcare perspective is applied, only costs accruing to the formal Dutch healthcare system will be included. To collect data on resource use, participants are asked to fill in cost questionnaires at baseline (T1), directly after delivery of the orthosis and at 3 and 6 months post orthotic treatment (T2 and T3). All cost categories will be valued in accordance with the Dutch manual for costing studies in health care[40].

Outcome measures used for the economic evaluation will be Quality Adjusted Life Years (QALYs), walking energy cost and GAS scores. QALYs will be based on the EQ-5D-5L, administered at each measurement point (T1, T2 and T3). The patients' EQ-5D-5L health states will be converted into utility scores using the Dutch tariff[41]. Subsequently, QALYs will be estimated by multiplying the patients' utility scores by the time spent in a certain health state.

Statistical analysis

Walking energy cost and secondary outcomes at each measurement point (T1, T2 and T3) will be analyzed with linear mixed models for repeated measurements to investigate the effectiveness of specialized orthotic care compared to usual orthotic care over time, adjusted for stratification and differences in baseline scores. Time and study group will be included as dependent variables. The differences in GAS scores between groups at 3 and 6 months follow-up will be tested with non-parametric Mann-Whitney Wilcoxon tests, as GAS scores are ordinal. All comparisons between groups are based on an intention-to-treat analysis.

For the economic evaluation, mean differences in total costs and effects between groups will be estimated using seemingly unrelated regression analyses. To account for the skewed nature of cost data, 95% confidence intervals surrounding cost differences will be estimated using Bias Corrected and Accelerated bootstrapping with 5000 replication. Subsequently, incremental cost-effectiveness ratios will be calculated by dividing the mean differences in costs by the mean differences in effects. To illustrate the joint uncertainty surrounding costs and effects, cost-effectiveness planes and cost-effectiveness acceptability curves will be plotted. In a cost-effectiveness acceptability curve, the probability of specialized orthotic care being cost-effective compared to usual orthotic care is plotted for a range of willingness to pay values (i.e. the amount of money decision-makers are willing to pay per unit of effect gained).

All statistical analyses will be performed using SPSS (V25; IBM SPSS, Chicago, Illinois, USA) and a statistical significance of $p < 0.05$ will be used in this study.

Patient and public involvement

Patients were actively involved in the preparation of this study through participating in meetings with the research group in the development stage of the study protocol and by providing feedback on the study procedures and the patient information documentation. During the conductance of the study,

For peer review only - http://bmjopen.bmj.com/site/about/guidelines.xhtml

patients will be informed about the progress and involved in patient recruitment and the interpretation, reporting and dissemination of the results.

For peer review only

DISCUSSION

The aim of this study is to examine the effectiveness and cost-effectiveness of specialized orthotic care compared to usual orthotic care on functioning in adults with slowly progressive NMD. This study has several strengths.

First, a broad range of outcome measures on consecutive time points before and after orthotic treatment will be collected. For the examination of effectiveness, we will not only analyze objective outcome measures, such as walking energy cost and gait biomechanics, but also patient-reported outcome measures, like satisfaction with the provided orthosis. Besides, personal treatment goals will be set at the activity and participation level of the ICF and are therefore considered to be highly relevant to the patient. By assessing outcome measures from multiple perspectives, we will be able to fully capture the functional effects of specialized orthotic care and, at the same time, gain insight in the underlying biomechanical working mechanisms. Additionally, compared to previous research, evidence will be obtained in a large group of adults suffering from leg muscle weakness, caused by many different slowly progressive neuromuscular disorders, which increases the generalizability of results.

Second, participants in the control group will be treated in a diverse sample of usual care centers in which leg orthoses are prescribed throughout the Netherlands. This allows for a broad comparison between specialized orthotic care and orthotic care as applied in current practice, which could be of importance for the improvement of overall quality and efficiency of orthotic care. On the other hand, treatment components of the usual care process are expected to differ amongst centers due to local policies and available facilities. This could complicate the identification of underlying mechanisms of action that could explain differences in treatment outcomes between groups. To enable cautious exploration of potential mechanisms that might lead to beneficial treatment outcomes, which could be useful for future implications, the process of usual orthotic care will be extensively documented.

Finally, a major strength is the economic evaluation that will be performed alongside the study. Since expensive orthotic devices increasingly become available due to technological developments, it is important to not only assess whether orthotic devices are effective, but also whether their additional health effects are worth their additional costs. This study will be the first to provide insights into the cost-effectiveness of orthotic care in NMD in general and of specialized orthotic care compared to usual orthotic care in particular.

In conclusion, this study aims to examine the effectiveness and cost-effectiveness of specialized orthotic care compared to usual orthotic care in adults with NMD. Insights could lead to improvements in the quality of orthotic care resulting in improvements in overall functionality of adults with NMD in daily living. Consequently, insights could lead to a more efficient use of already scarce (healthcare) resources.

For peer review only - http://bmjopen.bmj.com/site/about/guidelines.xhtml

ETHICS AND DISSEMINATION

The study protocol was approved by the Medical Ethics committee of the Amsterdam UMC, location Academic Medical Center (AMC), ABR-number 67268. The study is registered at the Dutch Trial Register (NL7511) and will be performed in accordance with good clinical practice guidelines. An independent monitor of the Amsterdam UMC will monitor the study multiple times during the conduction of the study. Aspects that will be monitored will include inclusion rate, trial master file, informed consent process, inclusion and exclusion criteria, randomization, trial procedures, source data verification, safety reporting and closing and reporting. The investigator will be encouraged to maintain the blinding as far as possible and code breaks should only occur in exceptional circumstances. Important protocol changes will be recorded (a new protocol version number will be assigned) and reported to the Medical Ethics Committee. When patients sign their informed consent, they will receive a participant ID, which will be coupled to all the data collected. Forms will be stored in a locked cabinet to assure anonymity. Only persons involved in the study have access to these forms. Insurance has been taken out for participation of patients in the study. After completion of the study, positive as well as negative or inconclusive results will be submitted to a peer-reviewed journal and presented at national and international scientific conferences. Furthermore, results will be disseminated through other media aimed at a broader audience including patients. The study data sets and codes of the data analysis are available on request. Furthermore, participants will be informed about the results of the study by means of a newsletter.

AUTHOR STATEMENT

M-AB, FSK and FN conceived the study. M-AB, FSK, FN, JMvD and EvD contributed to the study design and methods. M-AB, FSK, FN, JT, RL, VA and EvD participated in logistical planning of the study. EvD wrote the manuscript and is responsible for data acquisition and analysis. All authors conceived, provided feedback and approved the final version of the manuscript.

FUNDING

This study was supported by ZonMw, The Netherlands Organisation for Health Research and Development, grant number: 853001103. The funder will have no role in the execution, analyses, interpretation of the data of the study, or decision to submit results.

COMPETING INTERESTS

None declared.

For peer review only

For peer review only - http://bmjopen.bmj.com/site/about/guidelines.xhtml

REFERENCES

1. Demir YP. Neuromuscular Diseases and Rehabilitation. In: Suzuki T. Neurological Physical Therapy. BoD – Books on Demand 2017.
2. van der Krogt MM, Delp SL, Schwartz MH. How robust is human gait to muscle weakness? *Gait & posture* 2012;36(1):113-9. doi: 10.1016/j.gaitpost.2012.01.017 [published Online First: 2012/03/06]
3. Brehm M, Nollet F, Harlaar J. Energy demands of walking in persons with postpoliomyelitis syndrome: relationship with muscle strength and reproducibility. *Arch Phys Med Rehabil* 2006;87(1):136-40. doi: 10.1016/j.apmr.2005.08.123
4. Menotti F, Felici F, Damiani A, et al. Charcot-Marie-Tooth 1A patients with low level of impairment have a higher energy cost of walking than healthy individuals. *Neuromuscul Disord* 2011;21(1):52-7. doi: 10.1016/j.nmd.2010.09.008 [published Online First: 2010/11/16]
5. Abresch RT, Carter GT, Jensen MP, et al. Assessment of pain and health-related quality of life in slowly progressive neuromuscular disease. *Am J Hosp Palliat Care* 2002;19(1):39-48. doi: 10.1177/104990910201900109 [published Online First: 2002/08/14]
6. Bickerstaffe A, Beelen A, Nollet F. Circumstances and consequences of falls in polio survivors. *J Rehabil Med* 2010;42(10):908-15. doi: 10.2340/16501977-0620 [published Online First: 2010/10/30]
7. Pieterse AJ, Luttikhold TB, de Laat K, et al. Falls in patients with neuromuscular disorders. *Journal of the neurological sciences* 2006;251(1-2):87-90. doi: 10.1016/j.jns.2006.09.008 [published Online First: 2006/11/14]
8. Ahlstrom G, Sjoden PO. Coping with illness-related problems and quality of life in adult individuals with muscular dystrophy. *J Psychosom Res* 1996;41(4):365-76. doi: 10.1016/s0022-3999(96)00191-2 [published Online First: 1996/10/01]
9. Johnson NE, Heatwole CR, Dilek N, et al. Quality-of-life in Charcot-Marie-Tooth disease: the patient's perspective. *Neuromuscul Disord* 2014;24(11):1018-23. doi: 10.1016/j.nmd.2014.06.433 [published Online First: 2014/08/06]
10. Hsu JD, Michael JW, Fisk JR. Atlas of orthoses and assistive devices. In: Surgeons AAoO, ed. 4th edition ed. Philadelphia: American Academy of Orthopedic Surgeons 2008:299-309.
11. ISO. ISO 8549-1 - Vocabulary - Part 1: general terms for external limb prostheses and external orthoses. Genève: International Organization for Standardization, 1989.
12. Hijmans JM, Geertzen JH. Development of clinical guidelines for the prescription of orthoses in patients with neurological disorders in The Netherlands. *Prosthet Orthot Int* 2006;30(1):35-43. doi: 10.1080/03093640500533232 [published Online First: 2006/06/03]
13. Heim S, Jacobs NA, Jensen JS, et al. ISPO consensus conference on poliomyelitis. Consensus Statement and Syndicate Reports. International Society of Prosthetics and Orthotics 1997.
14. van der Wilk D, Dijkstra PU, Postema K, et al. Effects of ankle foot orthoses on body functions and activities in people with floppy paretic ankle muscles: a systematic review. *Clin Biomech (Bristol, Avon)* 2015;30(10):1009-25. doi: 10.1016/j.clinbiomech.2015.09.013 [published Online First: 2015/11/21]
15. McDaid C, Fayter D, Booth A, et al. Systematic review of the evidence on orthotic devices for the management of knee instability related to neuromuscular and central nervous system disorders. *BMJ Open* 2017;7(9):e015927. doi: 10.1136/bmjopen-2017-015927 [published Online First: 2017/09/08]
16. Brehm MA, Beelen A, Doorenbosch CA, et al. Effect of carbon-composite knee-ankle-foot orthoses on walking efficiency and gait in former polio patients. *J Rehabil Med* 2007;39(8):651-7. doi: 10.2340/16501977-0110 [published Online First: 2007/09/27]

For peer review only - http://bmjopen.bmj.com/site/about/guidelines.xhtml

17. Waterval NFJ, Brehm MA, Harlaar J, et al. Description of orthotic properties and effects evaluation of ankle-foot orthoses in non-spastic calf muscle weakness. *J Rehabil Med* 2020 doi: 10.2340/16501977-2642 [published Online First: 2020/01/15]
18. Brehm M, Nollet F. Beenorthesen bij neuromusculaire aandoeningen. Amsterdam: Reed business education 2014.
19. College van Zorgverzekeringen. Procesbeschrijving hulpmiddelenzorg. Den Haag: Nationaal ICT instituut in de Zorg 2009.
20. WHO. International classification of functioning, disability and health: ICF. Genève: World Health Organization, 2001.
21. ISO. ISO 9999 Assistive products for persons with disability – Classification and terminology. Draft International Standard. Genève: International Organization for Standardization, 2010.
22. Hebert JS. Ambulatory KAFOs: A Physiatry Perspective. *J Prosthet Orthot* 2006;18(7 Proceedings):169-74.
23. Brehm M, Bus SA, Harlaar J, et al. A candidate core set of outcome measures based on the International Classification of Functioning, Disability and Health for clinical studies on lower limb orthoses. *Prosthet Orthot Int* 2011;35(3):269-77. doi: 10.1177/0309364611413496 [published Online First: 2011/09/23]
24. Waterval NFJ, Nollet F, Harlaar J, et al. Modifying ankle foot orthosis stiffness in patients with calf muscle weakness: gait responses on group and individual level. *J Neuroeng Rehabil* 2019;16(1):120. doi: 10.1186/s12984-019-0600-2 [published Online First: 2019/10/19]
25. Healy A, Farmer S, Eddison N, et al. A scoping literature review of studies assessing effectiveness and cost-effectiveness of prosthetic and orthotic interventions. *Disabil Rehabil Assist Technol* 2019:1-7. doi: 10.1080/17483107.2018.1523953 [published Online First: 2019/01/18]
26. Vinci P, Gargiulo P. Poor compliance with ankle-foot-orthoses in Charcot-Marie-Tooth disease. *Eur J Phys Rehabil Med* 2008;44(1):27-31. [published Online First: 2008/04/04]
27. Swinnen E, Kerckhofs E. Compliance of patients wearing an orthotic device or orthopedic shoes: A systematic review. *J Bodyw Mov Ther* 2015;19(4):759-70. doi: 10.1016/j.jbmt.2015.06.008 [published Online First: 2015/11/26]
28. Chan AW, Tetzlaff JM, Altman DG, et al. SPIRIT 2013 Statement: Defining standard protocol items for clinical trials. *Ann Intern Med* 2013;158(3):200-207. doi: 10.7326/0003-4819-158-3-201302050-00583 [published Online First: 2013/01/09]
29. Council MR. *Aids to examination of the peripheral nervous system*. Memorandum no 45. London: Her Majesty's Stationary Office 1976.
30. Garby L, Astrup A. The relationship between the respiratory quotient and the energy equivalent of oxygen during simultaneous glucose and lipid oxidation and lipogenesis. *Acta physiologica Scandinavica* 1987;129(3):443-4. doi: 10.1111/j.1365-201x.1987.tb10613.x [published Online First: 1987/03/01]
31. Brehm MA, Verduijn S, Bon J, et al. Comparison of two 6-minute walk tests to assess walking capacity in polio survivors. *J Rehabil Med* 2017;49(9):732-37. doi: 10.2340/16501977-2264 [published Online First: 2017/09/21]
32. Kempen JC, Harlaar J, van der Kooi AJ, et al. Reliability of the walking energy cost test and the six-minute walk test in boys with Duchenne muscular dystrophy. *Neuromuscul Disord* 2014;24(3):216-21. doi: 10.1016/j.nmd.2013.11.015 [published Online First: 2013/12/25]
33. Steenbeek D, Ketelaar M, Galama K, et al. Goal attainment scaling in paediatric rehabilitation: a critical review of the literature. *Dev Med Child Neurol* 2007;49(7):550-6. doi: 10.1111/j.1469-8749.2007.00550.x [published Online First: 2007/06/27]
34. Steenbeek D, Meester-Delver A, Becher JG, et al. The effect of botulinum toxin type A treatment of the lower extremity on the level of functional abilities in children with cerebral palsy: evaluation with goal attainment scaling. *Clin Rehabil* 2005;19(3):274-82. doi: 10.1191/0269215505cr859oa [published Online First: 2005/04/30]
35. Dekkers K, de Viet E, Eilander H, et al. Goal attainment scaling (GAS) in de praktijk [Goal attainment scaling in clinical practice], 2011.

For peer review only - http://bmjopen.bmj.com/site/about/guidelines.xhtml

36. Gandek B, Ware JE, Jr., Aaronson NK, et al. Tests of data quality, scaling assumptions, and reliability of the SF-36 in eleven countries: results from the IQOLA Project. International Quality of Life Assessment. *J Clin Epidemiol* 1998;51(11):1149-58. doi: 10.1016/s0895-4356(98)00106-1 [published Online First: 1998/11/17]
37. Kempen GI, Yardley L, van Haastregt JC, et al. The Short FES-I: a shortened version of the falls efficacy scale-international to assess fear of falling. *Age Ageing* 2008;37(1):45-50. doi: 10.1093/ageing/afm157 [published Online First: 2007/11/23]
38. Krupp LB, LaRocca NG, Muir-Nash J, et al. The fatigue severity scale. Application to patients with multiple sclerosis and systemic lupus erythematosus. *Arch Neurol* 1989;46(10):1121-3. doi: 10.1001/archneur.1989.00520460115022 [published Online First: 1989/10/01]
39. Wessels RD, De Witte LP. Reliability and validity of the Dutch version of QUEST 2.0 with users of various types of assistive devices. *Disabil Rehabil* 2003;25(6):267-72. doi: 10.1080/0963828021000031197 [published Online First: 2003/03/08]
40. Kanters TA, Bouwmans CAM, van der Linden N, et al. Update of the Dutch manual for costing studies in health care. *PloS one* 2017;12(11):e0187477. doi: 10.1371/journal.pone.0187477 [published Online First: 2017/11/10]
41. Versteegh MM, Vermeulen KM, Evers SM, et al. Dutch Tariff for the Five-Level Version of EQ-5D. *Value health* 2016;19(4):343-52. doi: 10.1016/j.jval.2016.01.003 [published Online First: 2016/06/22]

For peer review only

For peer review only - http://bmjopen.bmj.com/site/about/guidelines.xhtml

Figure legends

***Figure 1**: Overview of the study design.*

For peer review only

For peer review only - http://bmjopen.bmj.com/site/about/guidelines.xhtml

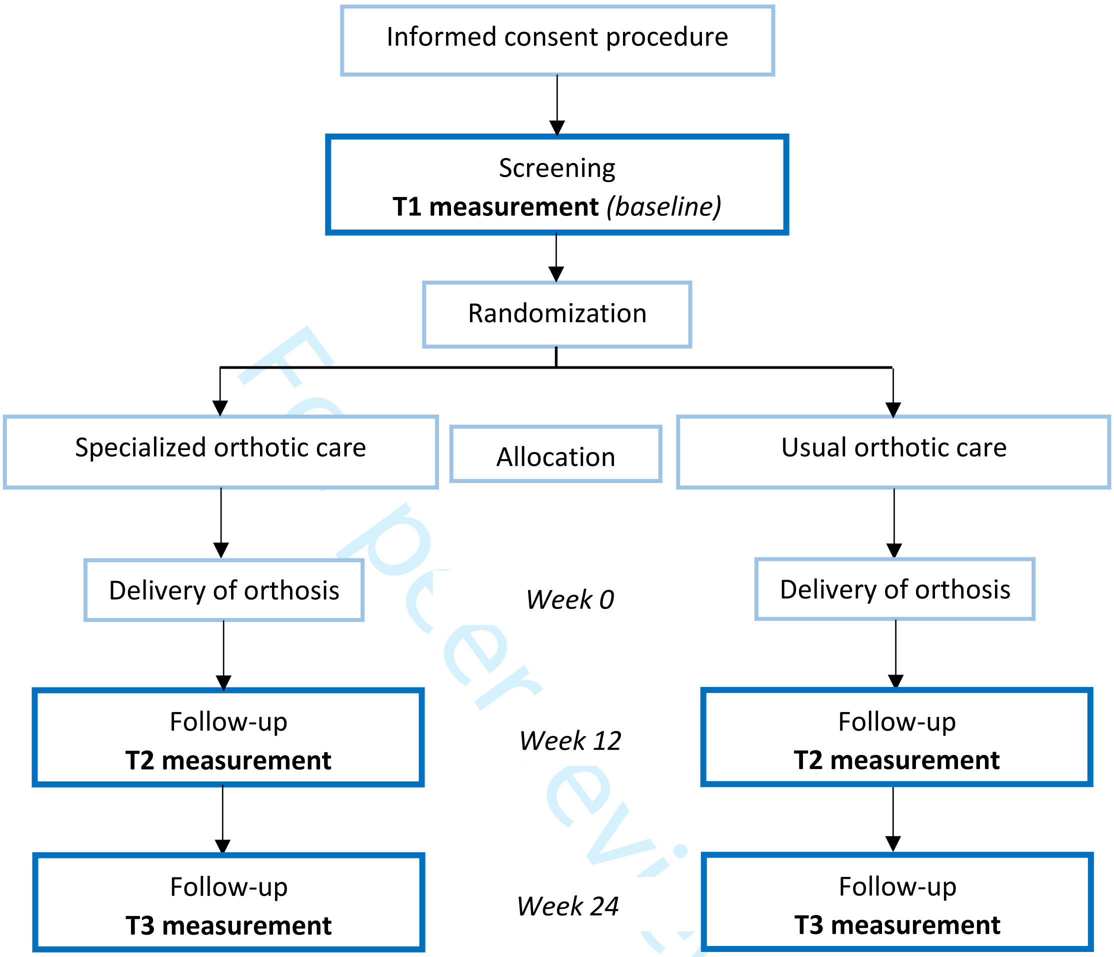
Informed consent procedure
Screening
T1 measurement *(baseline)*
Randomization
Specialized orthotic care
Allocation
Usual orthotic care
Delivery of orthosis
Week 0
Delivery of orthosis
Follow-up
T2 measurement
Week 12
Follow-up
T2 measurement
Follow-up
T3 measurement
Week 24
Follow-up
T3 measurement

Supplementary file 1:

Reporting checklist for protocol of a clinical trial.

Based on the SPIRIT guidelines.

		Reporting Item	Page Number
**Administrative information**			
Title	#1	Descriptive title identifying the study design, population, interventions, and, if applicable, trial acronym	1
Trial registration	#2a	Trial identifier and registry name. If not yet registered, name of intended registry	2
Trial registration: data set	#2b	All items from the World Health Organization Trial Registration Data Set	1-12
Protocol version	#3	Date and version identifier	1
Funding	#4	Sources and types of financial, material, and other support	12
Roles and responsibilities: contributorship	#5a	Names, affiliations, and roles of protocol contributors	1, 12
Roles and responsibilities: sponsor contact information	#5b	Name and contact information for the trial sponsor	1
Roles and responsibilities: sponsor and funder	#5c	Role of study sponsor and funders, if any, in study design; collection, management, analysis, and interpretation of data; writing of the report; and the decision to submit the report for publication, including whether they will have ultimate authority over any of these activities	12
Roles and responsibilities: committees	#5d	Composition, roles, and responsibilities of the coordinating centre, steering committee, endpoint adjudication committee, data management team, and other individuals or groups overseeing the trial, if applicable (see Item 21a for data monitoring committee)	12
**Introduction**			

For peer review only

Background and rationale	#6a	Description of research question and justification for undertaking the trial, including summary of relevant studies (published and unpublished) examining benefits and harms for each intervention	4
Background and rationale: choice of comparators	#6b	Explanation for choice of comparators	5
Objectives	#7	Specific objectives or hypotheses	5
Trial design	#8	Description of trial design including type of trial (eg, parallel group, crossover, factorial, single group), allocation ratio, and framework (eg, superiority, equivalence, non-inferiority, exploratory)	6
**Methods: Participants, interventions, and outcomes**			
Study setting	#9	Description of study settings (eg, community clinic, academic hospital) and list of countries where data will be collected. Reference to where list of study sites can be obtained	6
Eligibility criteria	#10	Inclusion and exclusion criteria for participants. If applicable, eligibility criteria for study centres and individuals who will perform the interventions (eg, surgeons, psychotherapists)	6
Interventions: description	#11a	Interventions for each group with sufficient detail to allow replication, including how and when they will be administered	7
Interventions: modifications	#11b	Criteria for discontinuing or modifying allocated interventions for a given trial participant (eg, drug dose change in response to harms, participant request, or improving / worsening disease)	n/a
Interventions: adherance	#11c	Strategies to improve adherence to intervention protocols, and any procedures for monitoring adherence (eg, drug tablet return; laboratory tests)	n/a
Interventions: concomitant care	#11d	Relevant concomitant care and interventions that are permitted or prohibited during the trial	n/a

For peer review only

Outcomes	#12	Primary, secondary, and other outcomes, including the specific measurement variable (eg, systolic blood pressure), analysis metric (eg, change from baseline, final value, time to event), method of aggregation (eg, median, proportion), and time point for each outcome. Explanation of the clinical relevance of chosen efficacy and harm outcomes is strongly recommended	8
Participant timeline	#13	Time schedule of enrolment, interventions (including any run-ins and washouts), assessments, and visits for participants. A schematic diagram is highly recommended (see Figure)	6
Sample size	#14	Estimated number of participants needed to achieve study objectives and how it was determined, including clinical and statistical assumptions supporting any sample size calculations	6
Recruitment	#15	Strategies for achieving adequate participant enrolment to reach target sample size	6
**Methods: Assignment of interventions (for controlled trials)**			
Allocation: sequence generation	#16a	Method of generating the allocation sequence (eg, computer-generated random numbers), and list of any factors for stratification. To reduce predictability of a random sequence, details of any planned restriction (eg, blocking) should be provided in a separate document that is unavailable to those who enrol participants or assign interventions	6-7
Allocation concealment mechanism	#16b	Mechanism of implementing the allocation sequence (eg, central telephone; sequentially numbered, opaque, sealed envelopes), describing any steps to conceal the sequence until interventions are assigned	6-7
Allocation: implementation	#16c	Who will generate the allocation sequence, who will enrol participants, and who will assign participants to interventions	6
Blinding (masking)	#17a	Who will be blinded after assignment to interventions (eg, trial participants, care providers, outcome assessors, data analysts), and how	7

For peer review only

Blinding (masking): emergency unblinding	#17b	If blinded, circumstances under which unblinding is permissible, and procedure for revealing a participant's allocated intervention during the trial	12
**Methods: Data collection, management, and analysis**			
Data collection plan	#18a	Plans for assessment and collection of outcome, baseline, and other trial data, including any related processes to promote data quality (eg, duplicate measurements, training of assessors) and a description of study instruments (eg, questionnaires, laboratory tests) along with their reliability and validity, if known. Reference to where data collection forms can be found, if not in the protocol	8-10
Data collection plan: retention	#18b	Plans to promote participant retention and complete follow-up, including list of any outcome data to be collected for participants who discontinue or deviate from intervention protocols	12
Data management	#19	Plans for data entry, coding, security, and storage, including any related processes to promote data quality (eg, double data entry; range checks for data values). Reference to where details of data management procedures can be found, if not in the protocol	12
Statistics: outcomes	#20a	Statistical methods for analysing primary and secondary outcomes. Reference to where other details of the statistical analysis plan can be found, if not in the protocol	10
Statistics: additional analyses	#20b	Methods for any additional analyses (eg, subgroup and adjusted analyses)	10
Statistics: analysis population and missing data	#20c	Definition of analysis population relating to protocol non-adherence (eg, as randomised analysis), and any statistical methods to handle missing data (eg, multiple imputation)	10
**Methods: Monitoring**			
Data monitoring: formal committee	#21a	Composition of data monitoring committee (DMC); summary of its role and reporting structure; statement of whether it is independent from the sponsor and competing interests; and reference to where further details about its charter can be	12

For peer review only

		found, if not in the protocol. Alternatively, an explanation of why a DMC is not needed	
Data monitoring: interim analysis	#21b	Description of any interim analyses and stopping guidelines, including who will have access to these interim results and make the final decision to terminate the trial	12
Harms	#22	Plans for collecting, assessing, reporting, and managing solicited and spontaneously reported adverse events and other unintended effects of trial interventions or trial conduct	6
Auditing	#23	Frequency and procedures for auditing trial conduct, if any, and whether the process will be independent from investigators and the sponsor	12
**Ethics and dissemination**			
Research ethics approval	#24	Plans for seeking research ethics committee / institutional review board (REC / IRB) approval	12
Protocol amendments	#25	Plans for communicating important protocol modifications (eg, changes to eligibility criteria, outcomes, analyses) to relevant parties (eg, investigators, REC / IRBs, trial participants, trial registries, journals, regulators)	12
Consent or assent	#26a	Who will obtain informed consent or assent from potential trial participants or authorised surrogates, and how (see Item 32)	6
Consent or assent: ancillary studies	#26b	Additional consent provisions for collection and use of participant data and biological specimens in ancillary studies, if applicable	n/a
Confidentiality	#27	How personal information about potential and enrolled participants will be collected, shared, and maintained in order to protect confidentiality before, during, and after the trial	12
Declaration of interests	#28	Financial and other competing interests for principal investigators for the overall trial and each study site	12
Data access	#29	Statement of who will have access to the final trial dataset, and disclosure of contractual agreements that limit such access for investigators	12

For peer review only

Ancillary and post trial care	#30	Provisions, if any, for ancillary and post-trial care, and for compensation to those who suffer harm from trial participation	12
Dissemination policy: trial results	#31a	Plans for investigators and sponsor to communicate trial results to participants, healthcare professionals, the public, and other relevant groups (eg, via publication, reporting in results databases, or other data sharing arrangements), including any publication restrictions	12
Dissemination policy: authorship	#31b	Authorship eligibility guidelines and any intended use of professional writers	12
Dissemination policy: reproducible research	#31c	Plans, if any, for granting public access to the full protocol, participant-level dataset, and statistical code	12
**Appendices**			
Informed consent materials	#32	Model consent form and other related documentation given to participants and authorised surrogates	supplement 2
Biological specimens	#33	Plans for collection, laboratory evaluation, and storage of biological specimens for genetic or molecular analysis in the current trial and for future use in ancillary studies, if applicable	n/a

The SPIRIT checklist is distributed under the terms of the Creative Commons Attribution License CC-BY-ND 3.0. This checklist was completed on 22. April 2020 using https://www.goodreports.org/, a tool made by the EQUATOR Network in collaboration with Penelope.ai

For peer review only

Subject information

Supplementary file 2: consent form subject

‘Specialized orthotic care for people with leg muscle weakness’

- I have read the information letter. I was also able to ask questions. My questions have been answered sufficiently. I have had enough time to decide whether or not to participate.

- I understand that participation is voluntary. I also know that I may decide at any time to not participate or to stop participating in the study. Without having to provide any reason.

- I give consent for my general practitioner to be informed of my participation in this study.

- I give consent to information being requested from my specialist(s) treating me about my medical history.

- I give consent to collect and use my data for answering the research question in this study.

- I know that for study monitoring purposes some individuals could have access to all my data. Those people are listed in this information letter. I consent to that access by these persons.

- I give consent for my general practitioner and/or treating specialist to be informed of unexpected findings which are (may be) of interest for my health.

- I **□ give**

  □ do not give

  consent for the further storage of my personal data for 15 years and retention for future research into the area of my disorder and the method of treatment.

- I **□ give**

  □ do not give

  consent to being contacted again after this study for a follow-up study.

For peer review only - http://bmjopen.bmj.com/site/about/guidelines.xhtml

Subject information

- I want to participate in this study.

Name of subject: ……………………………………………………………………………..
Signature: Date : __ / __ / __

I certify that I have fully informed this subject about the said study.

If information becomes known during the study that could influence the consent of the subject, I will inform him/her of this on time.

Name of investigator :…………………………………………………………………………
Signature: Date: __ / __ / __

The subject will receive a complete information letter, together with a signed version of the informed consent form.

For peer review only - http://bmjopen.bmj.com/site/about/guidelines.xhtml
